# Zona incerta dopamine neurons encode motivational vigor in food seeking

**DOI:** 10.1126/sciadv.adi5326

**Published:** 2023-11-17

**Authors:** Qiying Ye, Jeremiah Nunez, Xiaobing Zhang

**Affiliations:** Department of Psychology, Florida State University, Tallahassee, FL 32306, USA.

## Abstract

Energy deprivation triggers food seeking to ensure homeostatic consumption, but the neural coding of motivational vigor in food seeking during physical hunger remains unknown. Here, we report that ablation of dopamine (DA) neurons in zona incerta (ZI) but not ventral tegmental area potently impaired food seeking after fasting. ZI DA neurons and their projections to paraventricular thalamus (PVT) were quickly activated for food approach but inhibited during food consumption. Chemogenetic manipulation of ZI DA neurons bidirectionally regulated feeding motivation to control meal frequency but not meal size for food intake. Activation of ZI DA neurons promoted, but silencing of these neurons blocked, contextual memory associate with food reward. In addition, selective activation of ZI DA projections to PVT promoted food seeking for food consumption and transited positive-valence signals. Together, these findings reveal that ZI DA neurons encode motivational vigor in food seeking for food consumption through their projections to PVT.

## Teaser

Zona incerta dopamine neurons motivate food seeking triggered by energy deficit through projections to paraventricular thalamus.

## INTRODUCTION

Daily food intake is well controlled by the brain to sense the body’s energy states and then drive food-seeking behaviors that ensure homeostatic food consumption. It is relatively clear that both the hypothalamic arcuate nucleus (ARC) and the nucleus of the solitary tract (NTS) sense the body’s energy states to trigger or terminate homeostatic eating. However, it remains unknown how both homeostatic and satiety signals are integrated by the brain to drive and maintain food-seeking behaviors in particular during energy deprivation. Mice with global dopamine (DA) deficiency were reported to be aphagic with severe starvation (*1*). However, activation of ventral tegmental area (VTA) DA neurons or their projections to nucleus accumbens only increased meal frequency but not cumulative food consumption (*2*, *3*). Although VTA DA neurons may participate in some feeding-related behaviors especially hedonic eating, VTA DA signaling is not necessary for homeostatic eating especially food-seeking behaviors triggered by energy deficit (*4*). Therefore, it is critically important to decipher the neural coding of motivational vigor in food-seeking behaviors following central sensing of energy deficiency.

In addition to VTA, feeding-associated brain regions such as the hypothalamus and the zona incerta (ZI) also express DA neurons. We have reported that ARC DA neurons play an orexigenic role in energy homeostasis through inhibitory projections to both the paraventricular nucleus of hypothalamus and neighboring proopiomelanocortin neurons (*5*, *6*). However, it remains unknown about the function of ZI DA neurons and whether these neurons also regulate food intake though A13 DA neurons were identified in ZI for decades (*7*–*10*). ZI has been recently found to be a critical region for controlling motivated behaviors, especially feeding reported by our recent study and others (*11*–*19*). Early studies indicated that rostral ZI lesions led to decreased food intake (*20*). Previous studies also reported that ZI neurons responded to the sight and the approach of food and food teasing (*21*–*23*). These findings thus suggest that ZI neurons participate in regulating food seeking. Therefore, it is critical to know whether ZI DA neurons play a role for ZI in the regulation of food seeking triggered by energy deficit.

In this study, we used chemogenetics, optogenetics, fiber-photometry imaging, and selective cell ablation to investigate ZI DA neurons for their role in the regulation of feeding behavior. The results show that ablation of ZI, but not VTA, DA neurons impairs food-seeking behavior after fasting. Fiber-photometry results indicate that ZI DA neurons and their projections to paraventricular thalamus (PVT) are activated for approaching food but inhibited during food consumption. Chemogenetic activation of ZI DA neurons and optogenetic excitation of ZI-PVT DA projections promote food seeking and consumption, while chemogenetic silencing of these neurons decreases food-seeking behavior for food intake. Together, our findings support a critical role of ZI DA neurons in controlling motivational vigor for fasting-induced food seeking.

## RESULTS

### DA neurons in the ZI but not the VTA are required for maintaining motivational vigor in fasting-triggered food seeking

**Fig. 1.**
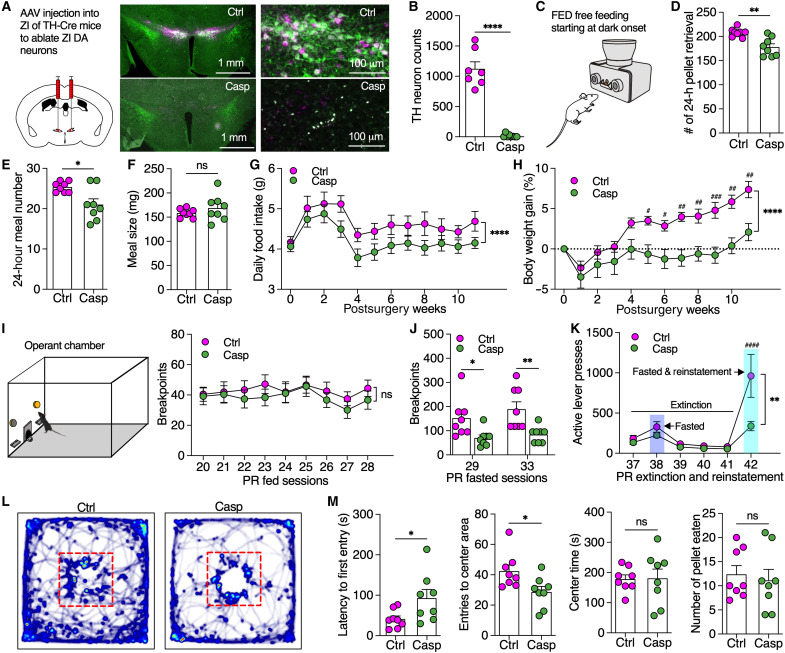
Selective ablation of ZI DA neurons impaired motivational vigor in fasting-triggered food seeking. (**A**) AAV was injected into bilateral ZI of TH-Cre mice to induce mCherry (*n* = 8 mice) or mCherry plus caspase expression (*n* = 8 mice) in ZI DA neurons. (**B**) TH-immunoreactive ZI neurons (green) were ablated by virus-induced caspase/mCherry (magenta) expression. (**C** to **F**) Meal pattern analysis using FED free feeding shows total number of pellet retrieval, meal numbers, and averaged meal size of 24 hours. (**G**) Daily food intake for both control and caspase mice for 11 weeks following virus injection. (**H**) Body weight gain for both control and caspase mice. (**I**) Breakpoint reached by fed mice during operant PR sessions of 45 min. (**J**) Breakpoints reached by 24-hour fasted mice. (**K**) Active lever presses during PR extinction and reinstatement sessions. (**L**) Real-time activity of mice in open-field chambers with food pellets placed in the center. (**M**) The latency to the first entry of the center area, the total entries to center area, the total time in the center area, and the number of food pellets consumed by mice in open-field chambers with food pellets placed in the center. Unaired *t* test for (B), (D), (E), (F), and (M), two-way ANOVA with post hoc Bonferroni test for (G) to (K).*P < 0.05, **P < 0.01, ***P < 0.001, ****P < 0.0001, and ^####^*P* < 0.0001; ns, no significance.

To revisit a role of VTA DA neurons in regulating food motivation, we injected adeno-associated virus (AAV) into VTA of TH-Cre mice to express caspase (Casp) for ablating VTA DA neurons (fig. S1, A and B). VTA DA ablation decreased daily meal numbers but increased meal size tested with feeding experimentation devices (FED) in home cages under free-feeding mode (fig. S1, C to F) (*24*) but had little effect on daily food consumption and body weight gain (fig. S1, G and H). Using operant behavioral tests under both fixed-ratio (FR) and progressive-ratio (PR) schedules of reinforcement (*25*), we found that mice with VTA DA ablation had significantly lower active lever presses during both FR and PR sessions and breakpoints for food reward during PR sessions under fed but not fasted conditions (fig. S1, I to L). This is consistent with previous findings that VTA DA neurons are involved in regulating motivation for palatable food intake in fed but not fasted states (*26*–*28*). To further reveal a source of DA neurons that are responsible for the DA control of homeostatic eating since mice with global DA knockout were aphagic (*1*), our attention was drawn to A13 DA neurons in the ZI, an area we recently reported to regulate feeding and body weight gain (*12*). We similarly injected AAV to selectively ablate ZI DA neurons in TH-Cre mice (Fig. 1, A and B). ZI DA ablation reduced meal frequency without an effect on meal size to decrease daily food intake and body weight gain (Fig. 1, C to H). Furthermore, we found that ablation of ZI DA neurons had no effect on active lever presses and breakpoints for food during both FR and PR sessions in fed mice (Fig. 1I and fig. S2, B to D). However, compared to control, mice with ZI DA ablation had a significantly lower active lever presses for breakpoints to obtain food during PR sessions after fasting of 24 hours (Fig. 1J and fig. S2E). ZI DA ablation also decreased active lever presses during reinstated PR sessions following extinction (Fig. 1K). In addition, mice with ZI DA ablation showed a decreased motivation for food under an anxiety-conflict condition with increased latency and decreased entries to the center of open-field chambers when food was placed in the center (Fig. 1, L and M, and fig. S3). Together, these findings indicated that DA neurons in ZI but not VTA are required for driving vigorous effort in food-seeking behaviors that ensure subsequent food consumption during energy deficiency.

### ZI DA neurons bidirectionally regulate motivation for food seeking to tune meal frequency for daily feeding control

**Fig. 2.**
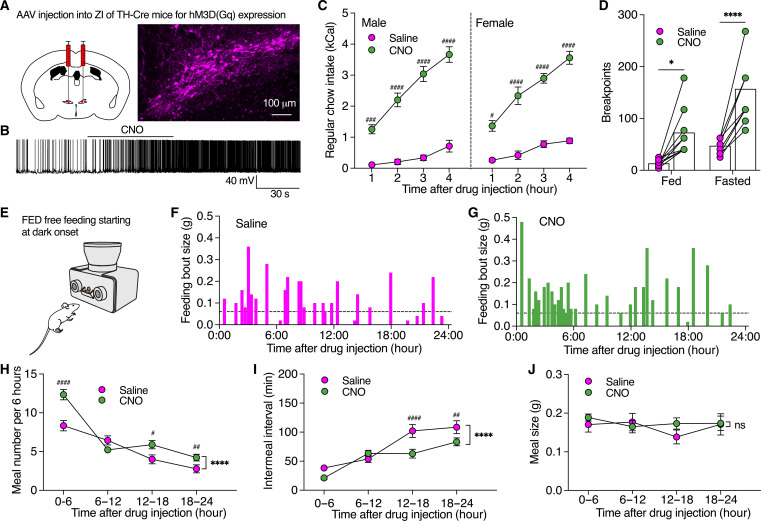
Chemogenetic activation of ZI DA neurons promoted motivational vigor in food seeking to regulate food consumption. (**A**) AAV was injected to bilateral ZI of TH-Cre mice to express excitatory hM3D(Gq) in ZI DA neurons. (**B**) A representative trace from slice recording shows that CNO (3 μM) excited an hM3D(Gq)-positive ZI DA neuron. (**C**) Regular food consumption during light cycle in both male (*n* = 12) and female (*n* = 8) TH-Cre mice with hM3D(Gq) expression in ZI DA neurons following IP injection of saline or CNO (2.0 mg/kg). (**D**) Breakpoints reached by both fed (*n* = 9) and partially fasted mice (70% of their daily food intake, *n* = 9) during PR sessions following IP injections of saline or CNO (2.0 mg/kg). (**E** to **G**) Real-time feeding bout sizes of a female ZI^TH-hM3D(Gq)^ mouse following IP injection of saline and CNO (2.0 mg/kg). FED devices at free-feeding modes were used for collecting real-time food intake in home cages. (**H** to **J**) Bar graphs showing meal numbers, intermeal intervals, and meal sizes of 6 hours. *n* = 9 female mice each group. Three-way ANOVA with post hoc Bonferroni test for (C), two-way repeated-measures (RM) ANOVA with post hoc Bonferroni test [(D) and (H) to (J)].

To determine how ZI DA neurons regulate feeding, we then injected AAV to express hM3D(Gq) selectively in ZI DA neurons of TH-Cre mice for chemogenetic activation (Fig. 2A and fig. S4). The function of hM3D(Gq) was confirmed with clozapine-N-oxide (CNO) (3.0 μM) using slice patch-clamp recordings (Fig. 2B). Chemogenetic activation of ZI DA neurons with intraperitoneal (IP) injection of CNO (2.0 mg/kg) in both sexes strongly increased regular food intake in both light and dark cycles and refeeding after 24-hour fasting (Fig. 2C and fig. S5, A and B). Electrophysiological data indicated that high-fat high-sucrose (HFHS) diet for 2 weeks depolarized the resting membrane potentials and increased the frequency of excitatory postsynaptic currents onto ZI DA neurons (fig. S6). Chemogenetic activation of these neurons also increased HFHS consumption in mice (fig. S5C). Furthermore, chemogenetic activation of ZI DA neurons with hM3D(Gq) expression potently promoted active lever presses to increase breakpoints for food reward of both light and dark cycles in both fed and fasted mice (Fig. 2D and figs. S7 and S8). However, CNO injection in control mice with mCherry expression in ZI DA neurons had no effect on food intake or operant lever presses during PR sessions (fig. S8). Using FED devices under both free-feeding mode and FR1 mode with nose pokes for food serving, we found that chemogenetic activation of ZI DA neurons increased daily meal numbers, decreased intermeal intervals, but did not affect meal size or meal duration (Fig. 2, E to J, and fig. S9). These results thus indicate that activation of ZI DA neurons promotes motivated effort to increase meal frequency for food intake especially under dark cycles and food-deprived conditions.

**Fig. 3.**
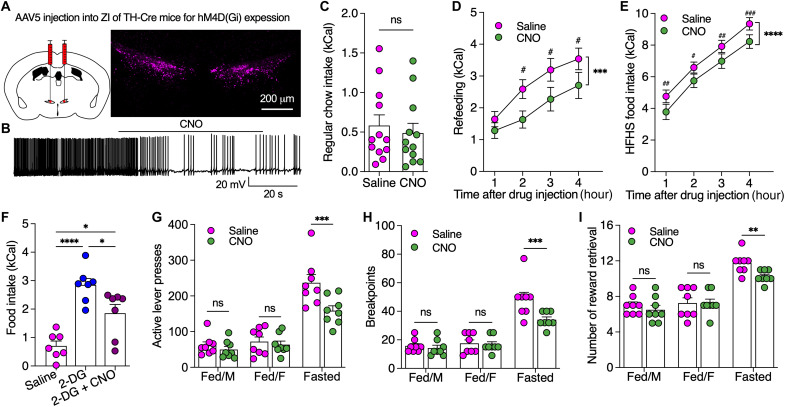
Chemogenetic inhibition of ZI DA neurons decreased motivated food seeking and consumption. (**A**) AAV was injected to bilateral ZI of TH-Cre mice to express inhibitory hM4D(Gi) in ZI DA neurons. (**B**) A representative trace shows that CNO (3.0 μM) inhibited a ZI^TH-hM4D(Gi)^ neuron. (**C**) Regular food intake of fed mice (*n* = 12) over 4 hours following saline and CNO injection. (**D**) Refeeding after 24 hours fasting following saline and CNO injection. (**E**) HFHS food intake following saline and CNO injection. (**F**) Food intake over 4 hours following injection of saline, 2-DG (200 mg/kg), or 2-DG plus CNO (2.0 mg/kg). *n* = 7 each group. (**G** to **I**) Bar graphs showing active lever presses, breakpoints, and number of rewards earned in both fed and fasted mice (70% of their daily food intake overnight) during operant PR tests of 45 min following saline or CNO (2.0 mg/kg) injection. Paired *t* test for (C), two-way RM ANOVA with post hoc Bonferroni test [(D), (E), and (G) to (I)], one-way ANOVA with post hoc Bonferroni test (F).

We further injected AAV to express hM4D(Gi) in ZI DA neurons of TH-Cre mice for chemogenetic inhibition (Fig. 3A). CNO (3.0 μM) hyperpolarized hM4D(Gi)-positive ZI DA neurons to decrease the neuronal activity (Fig. 3B). Chemogenetic inhibition of ZI DA neurons had little effect on regular food intake under fed conditions but decreased refeeding after 24-hour fasting and HFHS food intake (Fig. 3, C to E, and fig. S11, B and C). In addition, chemogenetic inhibition of ZI DA neurons impaired glucoprivic feeding evoked by IP injection of 2-deoxy-d-glucose (2-DG) (Fig. 3F) (*29*, *30*). Furthermore, we found chemogenetic inhibition of ZI DA neurons decreased active lever presses to reduce breakpoints for food reward primarily in fasted but not fed mice (Fig. 3, G to I). As a result, chemogenetic inhibition of ZI DA neurons decreased daily meal numbers but not meal size in mice (fig. S10). However, we did not observe CNO-induced reduction in refeeding of control mice with mCherry expression or effect of chemogenetic inhibition on normal locomotion (fig. S11). Together, these data indicate that inhibition of ZI DA neurons decreases motivational effort in food seeking to reduce food consumption selectively under fasted conditions, suggesting that ZI DA neurons participate in physiological feeding triggered by energy deficit.

### ZI DA neurons promote contextual memory for food seeking

**Fig. 4.**
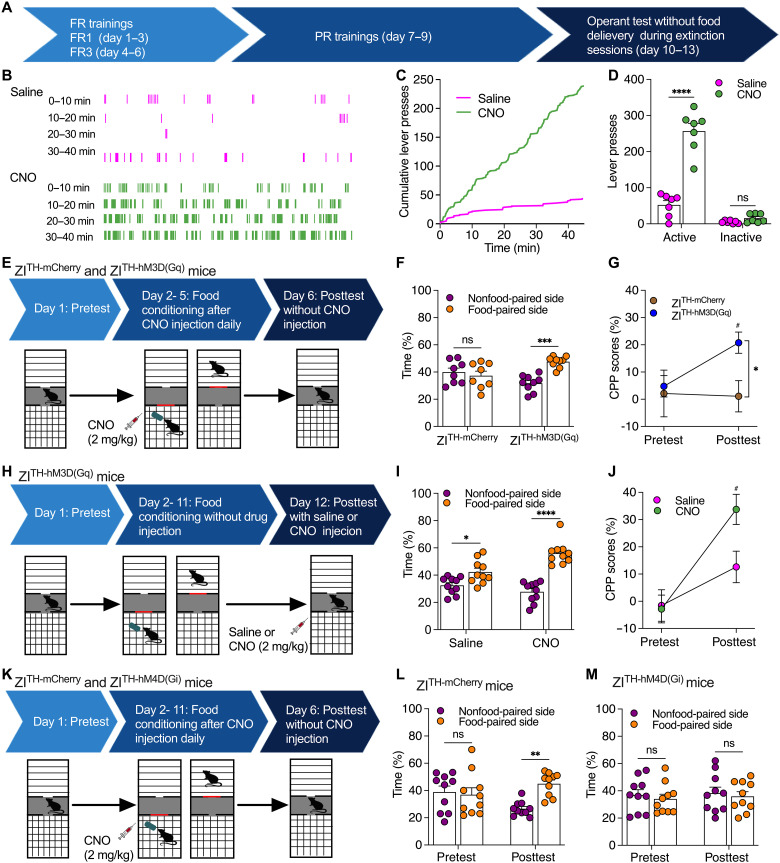
ZI DA neurons regulate both acquisition and expression of contextual food memory. (**A**) Timeline for operant training and tests. (**B**) Real-time lever presses during operant tests without reward delivery. (**C**) Cumulative lever presses during operant tests without reward delivery. (**D**) Presses of the food-paired active levers and the nonfood-paired inactive levers during operant tests without reward delivery. *n* = 7 mice each group. (**E**) Timeline for testing food CPP acquisition in both ZI^TH-mCherry^ (*n* = 8) or ZI^TH-hM3D(Gq)^ (*n* = 9) mice. (**F**) Percentage of time that mice spent in both nonfood-paired and food-paired sides during postconditioning sessions. (**G**) CPP scores during both preconditioning and postconditioning tests. (**H**) Timeline for testing food CPP expression. ZI^TH-hM3D(Gq)^ mice received 10-day place conditioning with HFHS food and pre- and postconditioning place preference tests. (**I**) Percentage of time that mice spent in both nonfood-paired and food-paired sides during postconditioning sessions following saline (*n* = 10 mice) or CNO (2.0 mg/kg, *n* = 10 mice) injection. (**J**) CPP scores in mice during both preconditioning and postconditioning tests. (**K**) Timeline for testing food CPP acquisition in both ZI^TH-mCherry^ (*n* = 10) or ZI^TH-hM4D(Gi)^ (*n* = 10) mice. (**L**) Percentage of time that control ZI^TH-mCherry^ mice spent in both nonfood-paired and food-paired sides during pre- and postconditioning tests. (**M**) Percentage of time that ZI^TH-hM4D(Gi)-mCherry^ mice spent in both nonfood-paired and food-paired sides during pre- and postconditioning tests. Two-way RM ANOVA with post hoc Bonferroni test for (D), (G), (J), (L), and (M). Two-way ANOVA with post hoc Bonferroni test for (F) and (I).

To determine whether ZI DA neurons drive food-seeking behaviors through promoting contextual memory associated with food reward, we tested operant behaviors during posttraining sessions without food delivery (Fig. 4A). Following the operant trainings, mice learned to press levers to obtain food reward. Although no pellet was delivered after lever presses during posttraining sessions, mice still compulsively pressed the lever that was paired to food reward previously. Chemogenetic activation of ZI DA neurons strongly increased compulsive lever presses for food seeking (Fig. 4, B to D).

We then asked whether ZI DA neurons regulate contextual food memory for food seeking, we performed a conditioned place preference (CPP) paradigm to test the preference to food-conditioned environment. Control ZI^TH-mCherry^ mice did not develop a preference for food-conditioned chambers after 4-day trainings when IP CNO (2 mg/kg) injection was given 30 min before food conditioning daily (Fig. 4, E to G). However, ZI^TH-hM3D(Gq)^ mice with CNO injection preferred food-paired chambers after 4-day food-paired trainings (Fig. 4, E to G). These data suggest that chemogenetic activation of ZI DA neurons promote acquisition of contextual food memory. We further used ZI^TH-hM3D(Gq)^ mice with longer food-paired trainings for 10 days to test a role of ZI DA neurons in expression of contextual food memory. All mice developed preferences for food-conditioned chambers after 10-day food-paired trainings (Fig. 4, H to J), while chemogenetic activation of ZI DA neurons during postconditioning sessions significantly increased the preference for food-conditioned chambers (Fig. 4, H to J). These data thus suggest activation of ZI DA neurons also promote expression of contextual food memory for food seeking, which is supported by previous findings that ZI integrates multiple sensory information for potential motivational drive (*31*).

To further reveal whether ZI DA neurons participate in the acquisition of contextual food memory for food seeking, we tested the effect of chemogenetic inhibition of ZI DA neurons on the development of CPP associated with food reward. Similar to the experimental design above, a longer food-paired training for 10 days was used to ensure that control ZI^TH-mCherry^ mice can develop contextual food memory (Fig. 4K). ZI^TH-mCherry^ mice spent significantly more time in food-paired compared to nonfood-paired compartment during posttest but not pre-test sessions (Fig. 4L), suggesting a development of place preference associated with contextual food memory in these mice. However, with daily chemogenetic inhibition of ZI DA neurons during food conditioning, ZI^TH-hM4D(Gi)-mCherry^ mice spent equally similar time in both nonfood- and food-paired compartment in both pretest and posttest sessions (Fig. 4M). These data thus indicate that inhibition of ZI DA neurons blocked the development of place preference associated with food conditioning, confirming that ZI DA neurons participate in developing contextual food memory for food seeking.

### ZI-PVT DA projections regulate food seeking and consumption

**Fig. 5.**
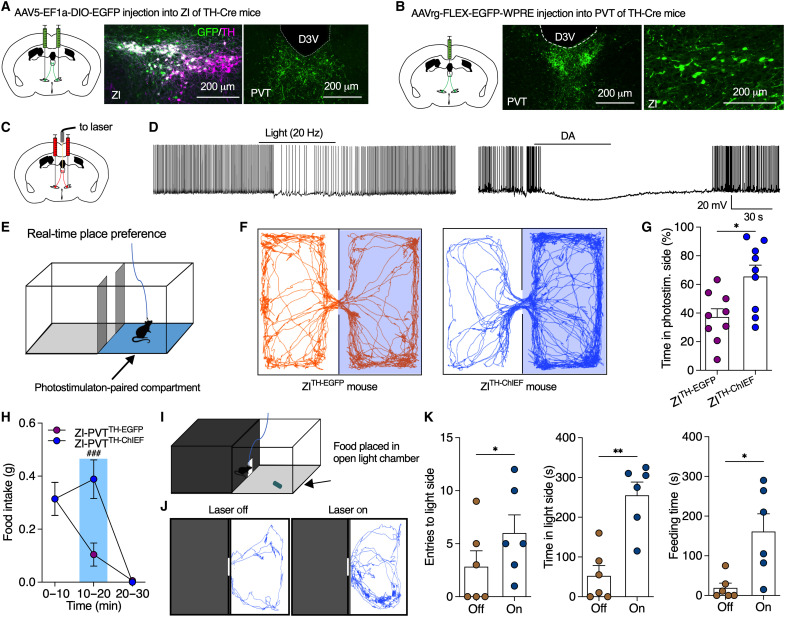
Optogenetic activation of ZI-PVT DA projections produced positive valence to promote food seeking for consumption. (**A**) AAV was injected into bilateral ZI of TH-Cre mice to induce EGFP expression in ZI DA neurons that sent EGFP-positive axons to PVT. (**B**) Retrograde AAV was injected into PVT of TH-Cre mice to trace ZI DA neurons that projected to PVT. (**C**) AAV was injected into bilateral ZI of TH-Cre mice to induce ChIEF or EGFP expression in ZI DA neurons. (**D**) Both photostimulation and DA (30 μM) inhibited PVT neurons surrounded by ChIEF-positive ZI DA axons. (**E**) A two-compartment chamber was used for real-time place preference test with one compartment paired with photostimulation (20 Hz). The laser was only turned on when mice entered the photostimulation-paired side. (**F**) Real-time motion of a control ZI^TH-EGFP^ and a ZI^TH-ChIEF^ mouse in the two-compartment chamber. (**G**) Percentage of time that mice spent in photostimulation-paired compartment. *n* = 9 mice each group. (**H**) HFHS food intake of mice before, during, and after PVT photostimulation. *n* = 7 mice each group. (**I**) A light/dark box with entrance connecting the two sides for food intake test over 10 min when food pellets were placed in the light side. (**J**) Real-time activity tracking in light side of the chambers during 10-min food intake test with or without photostimulation (20 Hz) of DA^ZI-PVT^ pathway. (**K**) Total entries to light side, cumulative time in light side, and cumulative feeding time during 10-min food intake. *n* = 6 mice per group. Unpaired *t* test for (G), two-way ANOVA with post hoc Bonferroni test for (H), and paired *t* test for (K).

ZI γ-aminobutyric acid (GABA) neurons send dense projections to PVT, an area that regulates behavioral motivation, for feeding control (*12*, *32*, *33*, *34*, *35*). Inactivation of PVT neurons also promoted place preference for sucrose seeking (*36*). To examine whether ZI DA neurons project to PVT for feeding regulation, we injected AAV to express enhanced green fluorescent protein (EGFP) in TH-positive ZI DA neurons of TH-Cre mice (Fig. 5A). EGFP-positive axons were detected in PVT, periaqueductal gray (PAG), and other areas (Fig. 5A and fig. S12). To further confirm ZI DA projections to PVT, we injected retrograde AAV into PVT of TH-Cre mice and found retrogradely labeled neurons in ZI (Fig. 5B). To identify a functional regulation of PVT neurons by ZI DA projections, we injected AAV to express ChIEF in bilateral ZI DA neurons of TH-Cre mice for slice recordings and behavioral tests with photostimulation of ChIEF-positive projections in PVT (Fig. 5C). Both photostimulation (20 Hz) and DA (30 μM) hyperpolarized and inhibited PVT neurons innervated by ChIEF-positive ZI DA terminals in the absence and presence of Bic (Fig. 5D and fig. S13, A to C). The outward currents evoked by photostimulation of ZI-PVT DA projections were blocked by D2 receptor antagonist sulpiride (fig. S13D), suggesting that postsynaptic D2 receptors in PVT neurons contribute to the inhibitory regulation of ZI-PVT DA pathways.

Activation of both lateral hypothalamic (LH) GABA neurons and VTA DA neurons was reported to produce positive valence for the regulation of food reward (*3*, *37*). Using self-photostimulation of ZI-PVT DA projections in two-compartment chambers for real-time place preference tests (Fig. 5E), we found that ZI^TH-ChIEF^ but not control ZI^TH-EGFP^ mice spent significantly longer time in photostimulation-paired side (Fig. 5, F and G) and continuously press levers to obtain self-stimulation of ZI-PVT DA pathways in operant chambers (fig. S14, A to D). ZI^TH-ChIEF^ mice still compulsively pressed the lever during the first two postpairing sessions without photostimulation following daily photostimulation-paired sessions of 5 days (fig. S14D). To further determine whether activation of ZI-PVT DA projections promotes positive valence for rewarding food memory, we tested photostimulation-evoked CPP expression (fig. S14, E and F). Before photostimulation pairing, mice spent less time in the compartments with smooth floor. However, mice developed and maintained a preference for smooth-floor compartments that were paired with photostimulation during the training sessions (fig. S14, G and H). Mice still spent more time in photostimulation-paired compartments during postpairing sessions 1 day after photostimulation-paired trainings for 3 days but gradually returned to pretest levels 3 days after paring (fig. S14, G and H). Together, these results indicate that activation of ZI-PVT DA projections produces positive valence and promotes rewarding food memory for food seeking.

Both optogenetic and chemogenetic activation of ZI DA axons locally in PVT increased food intake (Fig. 5H and fig. S15), while photostimulation of ZI-PAG DA projections produced little effect on both food intake and motivated food seeking (fig. S16). Light/dark boxes were previously used for testing feeding motivation in an anxiety-conflict environment (Fig. 5I) (*12*). Photostimulation of ZI-PVT DA projections significantly increased total entries to light compartment, total time spent in light side, and total feeding time (Fig. 5K). These results thus indicate that ZI-PVT DA activation promotes feeding motivation to increase food intake even under anxiety-associated conditions.

### ZI DA neurons respond to both food approach and consumption

**Fig. 6.**
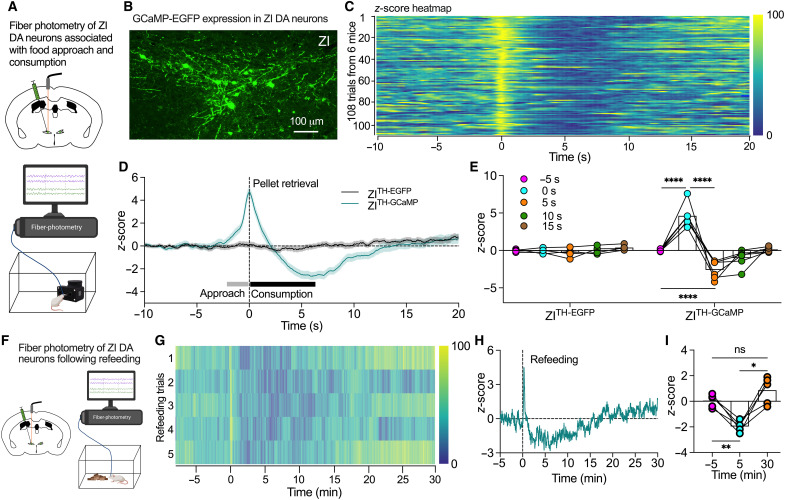
ZI DA neurons responded to food approach and consumption. (**A**) AAV was injected into ZI of TH-Cre mice to express GCaMP or EGFP in ZI DA neurons for fiber-photometry imaging of ZI DA neurons associated with food approach and consumption. (**B**) GCaMP7f was expressed in ZI DA neurons of TH-Cre mice. (**C**) Heatmap of normalized *z*-scores of ZI DA neuron activity from 108 feeding trials of ZI^TH-GCaMP^ mice (*n* = 6). (**D**) *z*-scores of ZI DA neurons aligned to the time of pellet retrieval from 85 feeding trials of control ZI^TH-EGFP^ mice and 108 trials of ZI^TH-GCaMP^ mice. (**E**) Increase in *z*-scores for food approach and decease during food consumption compared to 5 s before food retrieval in ZI^TH-GCaMP^ (*n* = 6) but not ZI^TH-EGFP^ mice (*n* = 5). Two-way RM ANOVA with post hoc Bonferroni test. (**F**) A diagram showing that AAV was injected into ZI of TH-Cre mice to express GCaMP in ZI DA neurons for fiber-photometry tests following refeeding. (**G**) Heatmaps of normalized *z*-scores of ZI DA neuron activity from refeeding trials of five fasted ZI^TH-GCaMP^ mice. (**H**) *z*-scores of ZI DA neurons aligned to the time of food delivery for refeeding from five fasted ZI^TH-GCaMP^ mice. (**I**) Bar graph with data plots showing the averages of *z*-score at the different time points before and after refeeding (*n* = 5 mice). One-way RM ANOVA with post hoc Bonferroni test.

Early studies have reported that sheep ZI neurons were activated responding to the approach of food (*21*, *22*). Neurons in the rostromedial ZI, the location of A13 DA neurons, were also activated by food teasing but not refeeding in fasted mice (*23*). These studies together suggest that ZI neurons are activated by food-associated signals to motivate food-seeking for food intake. To determine whether and how ZI DA neurons participate in the physiological feeding control, we applied in vivo fiber photometry to dynamically monitor the real-time activity of ZI DA neurons with AAV-induced GCaMP7f expression under a free-feeding test using FED devices (Fig. 6, A and B). We found that ZI DA neurons quickly increased their activity for approaching food and immediately decreased the activity after pellet retrieval followed by a rapid inhibition during food consumption before it went back to baseline level (Fig. 6, C to E). However, in control ZI^TH-EGFP^ mice, we observed no change of imaging signals in response to food approach and consumption (Fig. 6E and fig. S17). We further used the similar strategy to test whether ZI DA neurons have a long-lasting effect response to refeeding in fasted mice (Fig. 6F). We found the activity of ZI DA neurons were decreased during refeeding of about 15 min and then returned to the pre-refeeding level in 20 min after refeeding (Fig. 6, G to I). In control experiments with nonfood object delivery for those fasted mice, we did not observe any difference in signals among all these time points after the delivery (fig. S18). These data further suggest that ZI DA neurons respond to continuous food consumption after refeeding.

### ZI-PVT DA projections are activated for food approach but inhibited during food consumption

**Fig. 7.**
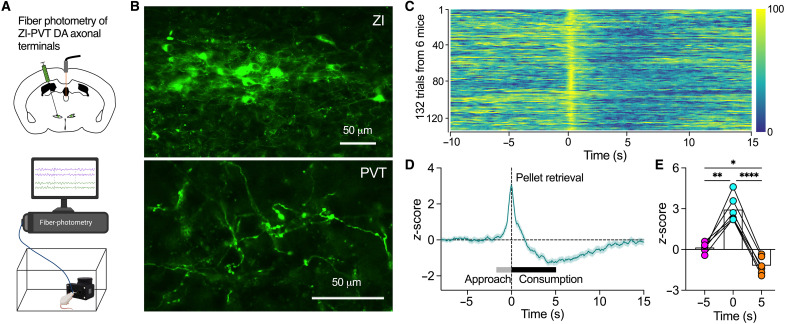
ZI DA axonal terminals in the PVT were activated for food approach but inhibited during food consumption. (**A**) AAV-hSynapsin1-FLEx-axon-GCaMP6s was injected into ZI of TH-Cre mice for fiber-photometry imaging of ZI DA axon terminals in PVT. (**B**) Representative images show GCaMP6s was expressed in both ZI DA somas (top) and axonal terminals in the PVT (bottom). (**C**) Heatmap of normalized *z*-scores of ZI-PVT DA axonal terminals from 132 feeding trials of six ZI^TH-GCaMP^ mice. (**D**) *z*-scores of ZI-PVT DA axonal terminals aligned to the time of pellet retrieval from 132 trials of six ZI^TH-GCaMP^ mice. (**E**) Bar graph with data plots showing the averages of *z*-score at the time of −5, 0, and 5 s (*n* = 6 mice). One-way RM ANOVA with post hoc Bonferroni test.

To further examine whether ZI-PVT axonal projections respond to feeding behavior including both food approach and consumption, we injected AAV-hSynapsin1-FLEx-axon-GCaMP6s to ZI of TH-Cre mice for expressing GCaMP6s in axons of ZI DA neurons for fiber-photometry imaging of ZI DA axons in PVT (Fig. 7, A and B). Similar to ZI DA somas, the activity of ZI DA axons in PVT also responded to food approach with quick increase but was decreased during food consumption (Fig. 7, C to E). These data further confirm that ZI-PVT projections are responsible for the control of ZI DA neuron in both food approach and consumption.

## DISCUSSION

In conclusion, we have shown that ZI DA neurons encode motivational vigor in fasting-triggered food seeking. Accordingly, ZI DA neurons are activated during energy deficit to promote both food motivation and contextual food memory that drive and maintain food-seeking behavior, which is necessary for hunger-triggered food consumption when vigorous effort is required for obtaining food. PVT is a critical postsynaptic target for ZI DA neurons to regulate food motivation and positive-valence signals for food seeking.

Positive valence systems are responsible for responses to positive motivational contexts, such as reward seeking, consummatory behavior, and reward learning. Both positive-valence signal and rewarding food memory play an important role in regulating food seeking and consumption. For instance, both LH GABA neurons and central amygdala 5-HT2A–expressing neurons modulate food consumption partially through a positive-valence mechanism for reinforcement of food reward (*38*–*40*). In addition, food conditioning develops contextual food memory that promotes food seeking under the conditions of energy deficit (*41*). In this study, we found activation of ZI DA neurons produced positive valence and promoted food-associated CPP, suggesting that ZI DA neurons regulate food seeking and consumption through a positive-valence mechanism that is probably responsible for food reward and contextual food memory. This is different from AgRP neurons that transit a negative-valence signal for energy sensing, which is modulated by food consumption and sensory food cues (*42*, *43*). In contrast to aphagia and severe starvation caused by loss of AgRP neurons (*44*), ablation of ZI DA neurons selectively impaired motivational vigor in food seeking to decrease meal frequency that reduce daily food intake and weight gain (Fig. 1). Activation of ZI DA neurons promoted food-seeking behaviors in both fed and fasted mice, while activating AgRP neurons had no effect on food seeking in fasted mice (*45*). These differences thus suggest that AgRP neurons are constantly activated by fasting for energy sensing but ZI DA neurons are only activated by environmental food cues for food seeking.

The present study also reveals a critical role of ZI DA neurons distinctly from VTA DA neurons in regulating feeding motivation. Activation of VTA DA neurons or their projections to nucleus accumbens had little effect on cumulative food intake though it increased meal frequency and promoted food motivation in fed animals (*2*, *3*). Consistent with these reports, our present data show that ablation of VTA DA neurons only reduced motivational vigor in food seeking in fed but not fasted mice. However, activation of ZI DA neurons increased feeding motivation in both fed and fasted mice, while ablation and silencing of these neurons reduced motivated food seeking selectively after fasting. The present findings thus demonstrate that ZI DA neurons drive food-seeking behaviors for homeostatic eating probably following metabolic sensing by other homeostasis-controlling neurons. In contrast, VTA DA neurons mainly regulate food reward for hedonic eating independent of physiological energy states. Together, these findings suggest that the brain uses two different DA pathways to drive hedonic and homeostatic eating. Although motivated food seeking is critical for ultimate food intake when vigorous effort is required for obtaining food, ZI DA neurons are not necessary for food consumption when food is freely available and food-seeking behavior is not needed. This is exactly what we observed in this study that ablation of ZI DA neurons decreased more than half of lever presses and breakpoints for food delivery under PR tasks in operant chambers (Fig. 1F). However, the daily food intake was slightly impaired by ablation of ZI DA neurons, although the decrease was still significant, when food was freely available in their home cages (Fig. 1D). These data further support that ZI DA neurons mainly regulate fasting-induced food seeking to control consequent food consumption.

ZI DA neurons are also distinct from LH GABA neurons, which are activated for appetitive or consummatory behaviors (*39*). Activation of ZI DA neurons strongly promoted motivated food seeking and consumption but not gnawing behaviors. However, activation of LH GABA neurons induced gnawing behaviors for food consumption (*46*, *47*). Activation of GABA^LH-VTA^ projections also induced compulsive eating but not reward seeking, while inhibition of these pathways did not reduce food intake in food-restricted mice (*47*). Together, these findings suggest that ZI DA neurons are responsible for driving and maintaining food-seeking behaviors, which is different from both LH GABA neurons for their role in driving consummatory behavior.

Our data also indicate that ZI DA neurons sent DA projections to inhibit PVT neurons for feeding control through activating inhibitory D2 receptors. Both ZI DA neurons and their axonal terminals in the PVT increased the activity for food approach but decreased activity during food consumption. The bidirectional response of ZI DA neurons and ZI-PVT DA axonal projections to food approach and consumption further supports a critical role of ZI DA signaling in driving food-seeking behavior for homeostatic consumption.

## MATERIALS AND METHODS

### Animals

TH-Cre mice were obtained from the Jackson Laboratory (Bar Harbor, ME, USA). TH-tdTomato mice were generated by Gensat/Rockefeller University and provided by A. van den Pol lab at the Yale University (*6*). All mice were housed in a climate-controlled vivarium on a 12:12-hour light/dark cycle and ad libitum access to food and water. All animals and experimental procedures in this study were approved by the Florida State University Institutional Animal Care and Use Committee. Both male and female mice used for this study were 8 to 10 weeks old at the beginning of the experiments.

### Intracerebral viral vector injections and optic fiber implantation

For chemogenetic activation or inhibition of ZI DA neurons, AAV5-hSyn-DIO-hM3D(Gq)-mCherry (Addgene, MA, USA), AAV5-hSyn-DIO-hM4D(Gi)-mCherry (Addgene, MA, USA), or AAV5-hSyn-DIO-mCherry (Addgene, MA, USA) was injected into bilateral ZI (0.2 μl per side) of TH-Cre mice. The stereotaxic coordinates for viral injections from Bregma were as follows: anterior-posterior (AP) = −1.3 mm, medial-lateral (ML) = ±0.5 mm, and dorsal-ventral (DV) = −4.5 mm from surface level of bregma. One month after viral injection, mice were used to for behavioral tests with chemogenetic modulation.

For optogenetic activation of ZI-PVT or ZI-PAG DA projections, AAV1-CAG-DIO-ChIEF-tdTomato (Signagen, MD, USA), AAV1-EF1a-DIO-ChR2(H134R)-EYFP-WPRE-HGHpA (Addgene, MA, USA), or AAV5-EF1a-DIO-EGFP (Addgene, MA, USA) was injected into bilateral ZI (0.2 μl per side) of TH-Cre mice. The stereotaxic coordinates for viral injections from Bregma were as follows: AP = −1.3 mm, ML = 0.5 mm, and DV = −4.5 mm from surface level of bregma. The optic fiber [outer diameter, 200 μm; numerical aperture (NA), 0.22; Doric Lenses, Canada) was implanted to target PVT or PAG. The stereotaxic coordinates for PVT were as follows: AP = −1.3 mm, ML = 0.05 mm, and DV = −2.8 mm from surface level of bregma. The stereotaxic coordinates for PAG were as follows: AP = −4.8 mm, ML = ±0.5 mm, and DV = −2.0 mm from surface level of Bregma. One month after viral injection, mice were used for behavioral tests with photostimulation of PVT or PAG.

For mapping ZI DA projections, AAV5-EF1a-DIO-EGFP (Addgene, MA, USA) was injected into bilateral ZI (0.2 μl per side) of TH-Cre mice. The stereotaxic coordinates for viral injections from bregma were as follows: AP = −1.3 mm, ML = ±0.5 mm, and DV = −4.5 mm from surface level of bregma. For retrograde neural tracing of ZI DA projections to PVT, AAVrg-CAG-FLEX-EGFP-WPRE (Addgene, MA, USA) was injected into PVT (0.2 μl) of TH-Cre mice. The stereotaxic coordinates for PVT were as follows: AP = −1.3 mm, ML = 0.05 mm, and DV = −3.0 mm from surface level of bregma. One month after viral injection, mice were perfused for anatomical imaging.

For fiber-photometry recording of ZI DA neurons, AAV5-syn-FLEX-jGCaMP7f-WPRE (Addgene, MA, USA) or AAV1-pCAG-FLEX-EGFP-WARE (Addgene, MA, USA) was injected into ZI (0.2 μl) of TH-Cre mice. The stereotaxic coordinates for viral injections from bregma were as follows: AP = −1.3 mm, ML = 0.5 mm, and DV = −4.5 mm from surface level of bregma. The optic fiber was implanted to target ZI of injection side. The stereotaxic coordinates for optic fiber implant from bregma were as follows: AP = −1.3 mm, ML = 0.5 mm, and DV = −4.5 mm from surface level of bregma. One month after viral injection, mice were used for photometry tests.

For fiber-photometry recording of ZI DA axons in the PVT, AAV-hSynapsin1-FLEx-axon-GCaMP6s (Addgene, MA, USA) was injected into ZI (0.2 μl) of TH-Cre mice. The stereotaxic coordinates for viral injections from bregma were as follows: AP = −1.3 mm, ML = 0.5 mm, and DV = −4.5 mm from surface level of bregma. The optic fiber was implanted to target PVT. The stereotaxic coordinates for optic fiber implant from bregma were as follows: AP = −1.3 mm, ML = 0.05 mm, and DV = −2.9 mm from surface level of bregma. One month after viral injection, mice were used for photometry tests.

For ablation of ZI and VTA DA neurons, 200 nl of AAV5-hSyn-DIO-mCherry [1:5 dilution in phosphate-buffered saline (PBS), control group] or AAV5-flex-taCasp3-TEVp mixed with AAV5-hSyn-DIO-mCherry (1:5 dilution in AAV5-flex-taCasp3-TEVp, Casp ablation group) was injected into bilateral ZI (coordinates: AP = −1.3 mm, ML = ±0.5 mm, and DV = −4.5 mm) or VTA (coordinates: AP = −3.1 mm, ML = ±0.4 mm, and DV = −4.5 mm) of TH-Cre mice. Mice were measured about daily food intake and weekly body weight before, during, and after injection surgery.

### Slice preparation and patch-clamp electrophysiology

Both male and female mice were used for preparing coronal brain slices (300 μm thick) containing the PVT as detailed in our previous studies (*6*, *12*). For whole-cell recording of PVT neurons, fresh brain slices were transferred to a recording chamber mounted on a Zeiss upright microscope (Zeiss, Berlin, Germany) and perfused with a continuous flow of gassed artificial cerebrospinal fluid solution containing 124 mM NaCl, 3 mM KCl, 2 mM MgCl_2_, 2 mM CaCl_2_, 1.23 mM NaH_2_PO_4_, 26 mM NaHCO_3_, and 10 mM glucose (gassed with 95% O_2_/5% CO_2_; 300 to 305 mOsm). Pipettes used for whole-cell recording had resistances ranging from 4 to 7 megohms when filled with pipette solution containing 145 mM potassium gluconate, 1 mM MgCl_2_, 10 mM HEPES, 1.1 mM EGTA, 2 mM Mg-ATP, 0.5 mM Na_2_-GTP, and 5 mM disodium phosphocreatine (pH 7.3 with KOH; 290 to 295 mOsm). The recording was performed at 33° ± 1°C using a dual-channel heat controller (Warner Instruments, Holliston, MA, USA). EPC-10 USB amplifier (HEKA Instruments, NY, USA) and PatchMaster 2x90.5 software (HEKA Elektronik, Lambrecht/Pfalz, Germany) were used to acquire and analyze the data. For voltage-clamp recording, the membrane potentials were held at −70 mV. Traces were processed using Igor Pro 6.37 (Wavemetrics, OR, USA). Spontaneous postsynaptic currents were analyzed with MiniAnalysis 6.03 (Synaptosoft Inc., GA, USA).

### Operant conditioning and progressive ratio schedules of reinforcement

Before operant conditioning training in mouse operant chambers (Med Associates, VT, USA), all C57BL/6J mice were food restricted (70% of their daily food intake) to facilitate the acquisition of lever-press responding until they learned to press the lever to obtain the food pellet in 3 to 5 days. Mice were provided their daily quota of food in the home cage after the termination of the training session. We used a program designed by our programing engineer for the data acquisition. During the training, mice were initially trained under FR1 sessions for 45 min daily. Animals had a choice between two press levers, an active lever press associated with a 3-s light cue, and a concomitant delivery of HFHS pellets (20 mg, 48.9% kcal as fat, Bio-Serv, NJ, USA) and an inactive lever press that remained inoperative and served as a control for general activity. Each active lever press triggered the delivery of one pellet during FR1 sessions. The lever remained inactive for 5 s after each food delivery so that mice were able to retrieve the pellet and supplementary presses during the inactive period did not drive food delivery. After a training period of about 7 to 10 days when three successive sessions of obtaining equal and more than 20 pellets during the FR1 session of 45 min, mice were then engaged in consecutive PR sessions of 45 min. For the PR session, the number of lever presses required for one food pellet delivery followed the order (calculated by the formula [5*e*^(R*0.2)^] − 5 where *R* is equal to the number of food rewards already earned plus 1): 1, 2, 4, 6, 9, 12, 15, 20, and so on (*25*). The maximal number of active lever presses performed to reach the final ratio was defined as the breakpoint, a value reflecting animals’ motivation to get the food reward. During PR extinction sessions, all lever presses were inactive to trigger any cue response or food delivery.

### Food intake in home cages

Regular chow intake over 4 hours was measured daily from 11:00 a.m. to 3:00 p.m. for light cycles and 7:00 p.m. to 11:00 p.m. for dark cycles in their home cages. When the daily regular chow intake over 4 hours was relatively stable for at least three successive days, mice were ready for food intake tests with drug treatment. On test days, regular chow or HFHS food intake was measured 45 min after IP drug infusion. In some experiments, mice were fasted for 24 hours before food intake tests.

### Food intake test using FED for meal pattern analysis

FED, an open-source home-cage compatible device, was used for food intake test and operant behavior for food delivery (*24*). Mice were singly housed, and the FED was placed in their cages for 6 days on a 12/12 on/off light cycle. For the free-feeding mode, a 20-mg pellet (Bio-Serv, NJ, USA) was dispensed into a feeding well of the FED device and monitored with a beam break. When the pellet was removed, the timestamp of removal and the latency to retrieve the pellet were logged to the internal microSD card and a new pellet was dispensed. For FR1 mode, FED logs the timestamps of each “nose-poke” event to the internal storage. When the mouse activated the left nose-poke the FED delivered a combined auditory tone (4 kHz for 0.3 s) and visual [all eight light-emitting diodes (LEDs) light in blue] stimulus and dispensed a pellet. While the pellet remained in the well both pokes remain inactive to prohibit multiple pellet delivery into the well. When the pellet was removed, the timestamp of removal and the latency to retrieve the pellet were logged. For chemogenetic manipulation of ZI DA neurons, saline, or CNO (2.0 mg/kg) was injected at 19:00 immediately before dark cycle of the experimental day. Food intake was recorded for meal pattern analysis. For meal pattern analysis, a feeding bout was defined as a meal if ≥60 mg of food was ingested and if it was separated from another meal by ≥10 min (*48*).

### Dark/light conflict test with food

Mice were exposed to HFHS food pellets 2 hours daily, for at least 3 days, before food intake was tested in the light/dark box where HFHS pellets were placed in the light compartment. At the beginning of the test, mice were placed in the light compartment, facing the entry to the dark compartment. A digital camera over the light/dark box was used to track and record the activity of mice in the light compartment. Latency to first exit, total entries to the light compartment, percentage of time spent in the light compartment, food approaches, and food intake were recorded and measured in 10-min tests with or without photostimulation.

### Open-field tests with and without food in the center area

Mice were fasted for 24 hours before the test with free access to water. On the test day, all mice were placed into the same corner of an illuminated open field (50 cm by 50 cm by 38 cm) with an opaque, white acrylic floor. Food pellets were placed in a cup in the center of the open field and all mice were allowed to explore freely. Real-time activity of mice in the chambers were videotaped for data analysis using Ethovision XT video tracking and analysis software (Noldus, Wageningen, The Netherlands). Latencies to the first entry to the center area, total entries to the center area, and total time spent in the center area (25 cm by 25 cm) were calculated with a limit of 10 min. For normal locomotion tests, ad lib mice were tested for 10 min in the open-field chambers without food. Both distance traveled and velocity of mice movement during the 10-min tests were analyzed for determining the locomotor activity. After the tests, mice were removed from the open field and the numbers of pellet eaten were immediately counted.

### Operant lever presses for self-photostimulation

At least 1 month after viral vector injection to induce ChIEF-tdTomato expression in ZI DA neurons of TH-Cre mice, mice were placed in an operant chamber (Med Associates, VT, USA) for self-photostimulation of PVT. Mice received photostimulation of 3 s immediately after every lever press. Continuous lever presses led to cumulative photostimulation. A digital camera was used to record the mouse movement track and behaviors during 30-min daily sessions for 9 days.

### Real-time place preference with photostimulation

At least 1 month after viral vector injection to induce ChIEF-tdTomato expression in ZI DA neurons of TH-Cre mice, TH-Cre mice were tested for real-time place preference in a two-compartment chamber lacking additional contextual cues. One compartment was paired with a 20-Hz photostimulation and the other identical compartment was without photostimulation. Total test duration was 10 min.

### CPP paired with photostimulation

At least 1 month after viral vector injection to induce ChIEF-tdTomato expression in ZI DA neurons of TH-Cre mice, TH-Cre mice were first pretested for place preference in a two-compartment chamber with rough wire floor for one compartment and smooth floor for another compartment for 10 min. In the next 3 days following pretest, mice were trained for 10 min daily with photostimulation of PVT when they entered and stayed in the compartment with smooth floor. After photostimulation-paired training for 3 days, mice then received postpairing place preference tests without photostimulation of 10 min daily for another 3 days. A digital camera was used to record the mouse movement track during the 10-min test daily.

### CPP paired with HFHS food

A 70 cm by 24 cm rectangular Plexiglass chamber with three compartments separated by removable Plexiglass walls was used for these experiments. The left and right compartments (28 cm by 24 cm each) had distinct wall patterns (black and white stripes versus white with black circles) and flooring (smooth versus rough wire floors). The center compartment measured 11.5 cm by 24 cm with no wall patterns and a smooth clear floor.

For acquisition of HFHS-associated place preference, mice first received pretest for baseline preference. Subjects were placed in the center compartment and then the barriers were lifted that allowed the subject to explore the entire apparatus for 10 min. On days 2 to 5 for experiments with chemogenetic activation or days 2 to 11 for experiments with chemogenetic inhibition, two conditioning sessions were conducted per day, separated by at least 3 hours. In the first session, mice were confined to one side compartment that contained a HFHS pellet for 30 min. Saline or CNO (2.0 mg/kg) was injected intraperitoneally 30 to 45 min before HFHS conditioning. In the second session, mice were confined to the opposite side compartment without food for another 30 min. On day 6 for experiments with chemogenetic activation or day 12 for experiments with chemogenetic inhibition, postconditioning tests were conducted in the same manner as the baseline preference test.

For the expression of HFHS-associated place preference, mice also received pretest for baseline preference for 10 min similar to acquisition experiments above. On days 2 to 11, two conditioning sessions were conducted per day, separated by at least 3 hours. In the first session, mice were confined to one side compartment that contained HFHS pellets for 30 min; in the second session, mice were confined to the opposite compartment without food for 30 min. HFHS-paired side was counterbalanced in each group. On day 12, postconditioning tests were conducted in the same manner as baseline preference tests. Thirty min before postconditioning tests, saline or CNO (2.0 mg/kg) was injected intraperitoneally.

### In vivo fiber photometry

Four weeks after virus injection and fiber optic implantation, a fiber photometry system (R810, RWD Life Science Co. Ltd., China) was used for recording fluorescence signal (GCaMP and isosbestic wavelengths), which was produced by an excitation laser beam from 470- and 410-nm LED lights. Calcium fluorescence signal were acquired at 60 Hz with alternating pluses of both lights. The power at the end of the optical fiber (200 μm and 0.37 NA) was adjusted to 30 to 40 μW. Recording parameters were set based on pilot studies that demonstrated the least amount of photobleaching, while allowing for the sufficient detection of the calcium response. A digital camera was used for behavioral recordings that were synchronized with calcium signal recordings.

To monitor the calcium signals of neurons associated with feeding behaviors, a FED3 device was used for the delivery of food pellet using a free-feeding mode. To minimize novelty-caused stress, mice were habituated for several days to the FED3 devices using the free-feeding mode. Before fiber photometry recordings, mice were fasted for 24 hours to increase feeding motivation for the testing. On the experimental day, mice were allowed to acclimate in the home cage for 30 min with connection to the fiber photometry system. To better monitor the response of ZI DA neurons to food approach and consumption during a 30-min session, the FED3 device was set to deliver one 20-mg pellet (Bio-Serv, NJ, USA) 60 s after every previous pellet was retrieved by mice. For continuous refeeding test, regular chow was directly delivered to the testing chamber during fiber-photometry recordings. For control tests with nonfood object, an object with a shape and size similar to chow pellets was delivered into the testing chambers during recording.

Regarding quantification, the filtered 410-nm signal was aligned with the 470-nm signal by using the least-square linear fit. *z*-scores were calculated according to (470-nm signal-fitted 410-nm signal)/(fitted 410-nm signal). Normalized *z*-scores were obtained when the largest value of each trial was set to 100% and smallest value was defined as 0% using GraphPad Prism 9.

### Immunocytochemistry

Mice were anesthetized with ketamine, and then perfused transcardially with saline followed by 4% paraformaldehyde or 3% glutaraldehyde. The 30-μm-thick coronal sections were cut on a cryostat, immersed in PBS for 1 hour, treated with 2% normal horse serum in PBS for 1 hour, and then incubated overnight at 4°C in polyclonal rabbit TH antibody (1:2000; AB152, Millipore) (*5*, *6*) or anti-DA antiserum (1: 1000, from H. Steinbusch at the Maastricht University) described in detail elsewhere (*49*). After washing in PBS, sections were placed in secondary Alexa Fluor 488–conjugated (JacksonImmunoResearch, 711-545-152) or Alexa Fluor 594–conjugated donkey anti-rabbit IgG (Jackson ImmunoResearch, 711-585-152) at a dilution of 1:500 for 4 hours, washed, and mounted on glass slides for imaging under microscope.

### Optogenetic stimulation

Light was delivered through a fiber optic cable and into the brain of mice with a fiber optic implant using a blue-light laser (473 nm; Laserglow Technologies, Canada). Mice were briefly anesthetized using isoflurane to allow connection of the cable to the implant. Stimulation pulses (10 ms) of 20 Hz were controlled by a pulse generator (Doric Lenses, Canada) and delivered to stimulate ZI DA terminals in the PVT. During slice recordings, 20-Hz photostimulation was also delivered to target the PVT of brain tissues in the recording chamber.

### Overall experimental design and statistical analysis

Animals were randomly assigned to different experimental groups before testing. Data collection and analysis were not performed blind to the conditions of the experiments. No statistical methods were used to predetermine sample sizes; our sample sizes are similar to those generally used in the field. Data are expressed as mean ± SEM. Sample sizes (*n* number) refer to values obtained from individual animal in all behavioral experiments and individual cell recordings. Statistical analyses were performed using GraphPad Prism 9.0. Statistical significance was taken as *P < 0.05, **P < 0.01, ***P < 0.001, and ****P < 0.0001, as determined by one-way, two-way, three-way analysis of variance (ANOVA) followed by post hoc Bonferroni test, two-tailed paired, or unpaired *t* test.
