# Supplementary material for: Zona incerta dopamine neurons encode motivational vigor in food seeking
Source: Sci Adv. 2023 Nov 17;9(46):eadi5326. doi: 10.1126/sciadv.adi5326 (PMC10656063; doi:10.1126/sciadv.adi5326)
Supplement: Supplementary file 1 — Figs. S1 to S18 [file sciadv.adi5326_sm.pdf]

Science Advances
AAAS

Supplementary Materials for

Zona incerta dopamine neurons encode motivational vigor in food seeking

Qiying Ye *et al.*

Corresponding author: Xiaobing Zhang, xzhang@psy.fsu.edu

Sci. Adv. **9**, eadi5326 (2023)
DOI: 10.1126/sciadv.adi5326

This PDF file includes:

Figs. S1 to S18

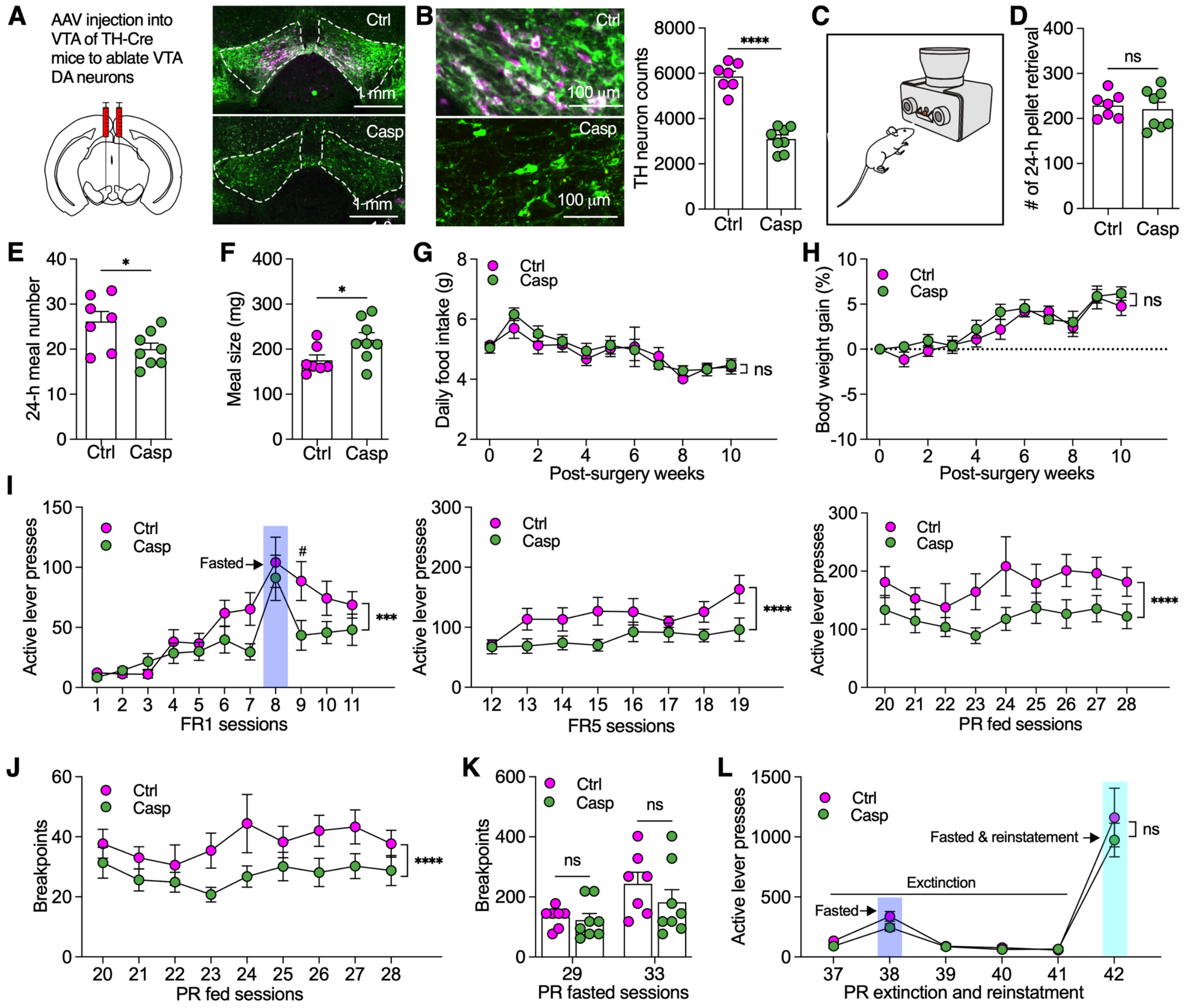

Fig. S1: Selective ablation of VTA DA neurons reduced the motivational effort for seeking food reward at fed but not fasted states. (**A**) AAV was injected into VTA of TH-Cre mice to induce mCherry (magenta) or mCherry plus caspase expression selectively in VTA DA neurons. (**B**) TH-immunoreactive VTA neurons (green) were ablated by virus-induced caspase expression. n= 7 mice for each group. Unpaired t test. (**C-F**) Meal pattern analysis obtained from home-cage free feeding using FED devices shows total number of pellet retrieval, meal numbers, and averaged meal size of 24 h. n= 7 for ctrl and n= 8 for Casp group. Unpaired t test. (**G, H**) Daily food intake and body weight gain for both groups (n= 7 for ctrl and n= 8 for Casp group) for 11 weeks following virus injection. (**I**) Active lever presses of mice during FR1, FR5 and PR fed sessions of 45 min. Two-way ANOVA with Post hoc Bonferroni test. (**J**) Breakpoint reached by fed mice (n= 7 for ctrl and n= 8 for Casp group) during operant PR sessions of 45 min. Two-way ANOVA with Post hoc Bonferroni test. (**K**) Breakpoints reached by fasted mice (n= 7 for ctrl and n= 8 for Casp group). Two-way ANOVA with Post hoc Bonferroni test. (**L**) Active lever presses at fed and fasted conditions (n= 7 for ctrl and n= 8 for Casp group) during PR extinction and reinstatement sessions. Two-way ANOVA with Post hoc Bonferroni test.

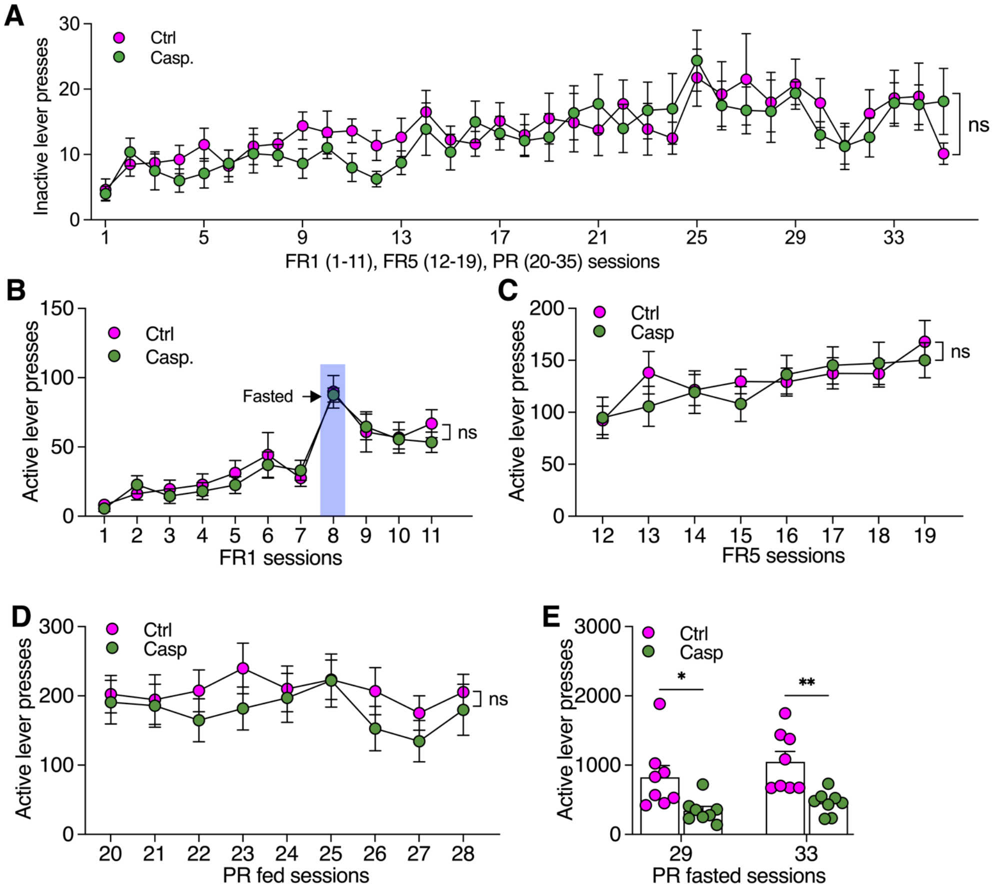

Fig. S2: (**A**) Inactive lever presses of mice during FR1, FR5, and PR fed sessions of 45 min. Two-way ANOVA with Post hoc Bonferroni test. (**B-E**) Active lever presses of mice during FR1, FR5, and PR fed and fasted sessions of 45 min. Two-way ANOVA with Post hoc Bonferroni test.

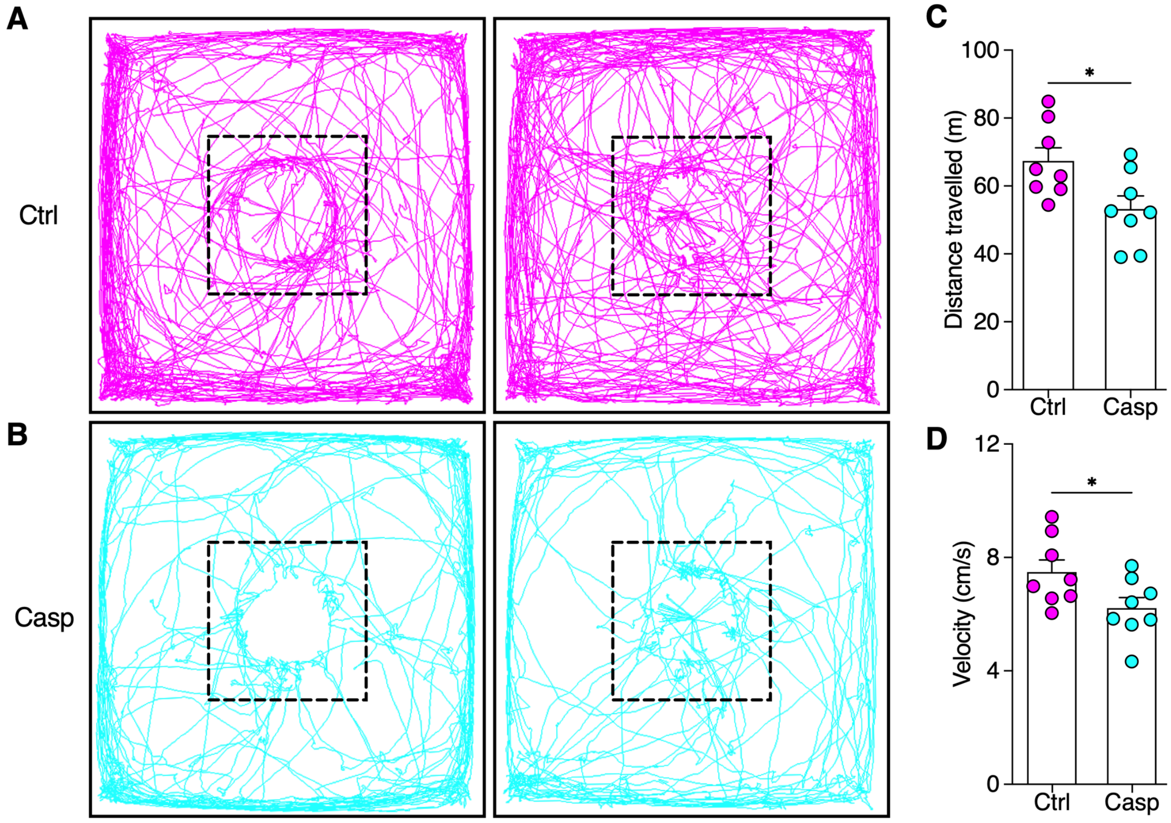

Fig. S3: (**A**) Real-time activity of two control mice in open-field chambers with food pellets placed in the center. n= 7 mice each group. (**B**) Real-time activity of two Casp mice in open-field chambers with food pellets placed in the center. (**C**) Total of distance travelled in open-field chambers. n= 8 mice each group. Unpaired t test. (**D**) velocity of mice travelled in open-field chambers. n= 8 mice each group. Unpaired t test.

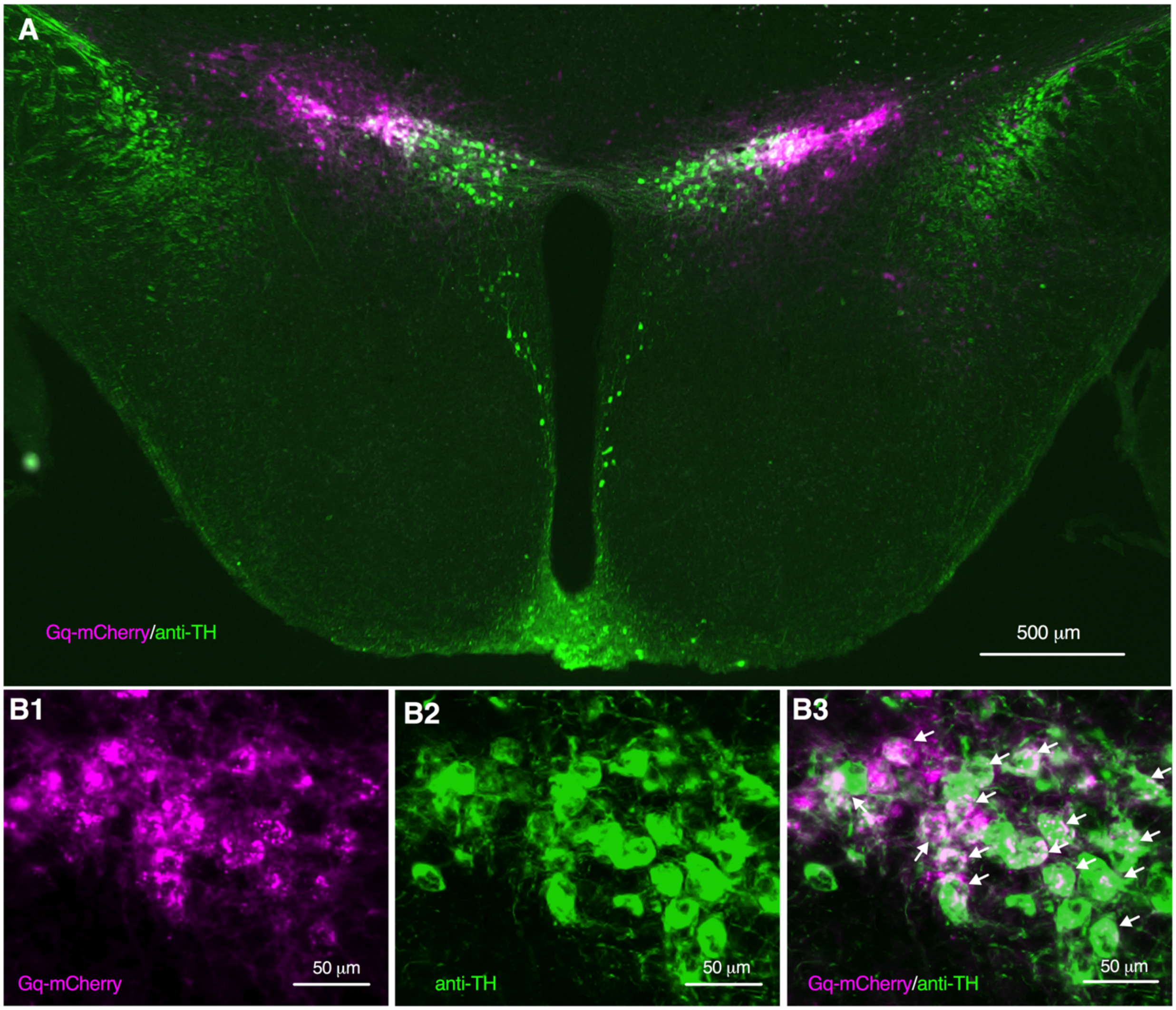

Fig. S4: Excitatory DREADDS hM3D(Gq) was expressed in ZI TH neurons. (**A**) A fluorescent image showing both hM3D(Gq)-mCherry (pink) and anti-TH immunoreactivity (green) in ZI neurons of mice with viral injection. (**B1-B3**) Zoomed-in images showing co-expression of hM3D(Gq)-mCherry and TH in ZI neurons.

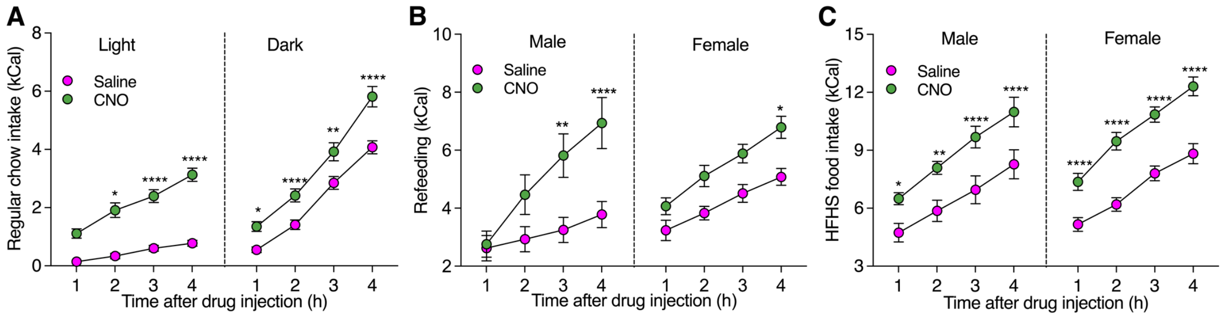

Fig. S5: Chemogenetic activation of ZI DA neurons increased food intake of both male and female mice in different conditions. (**A**) Regular food in both light and dark cycles. (**B**) Refeeding after 24 h fasting. (**C**) HFHS food intake. Three-way RM ANOVA with Post hoc Bonferroni test.

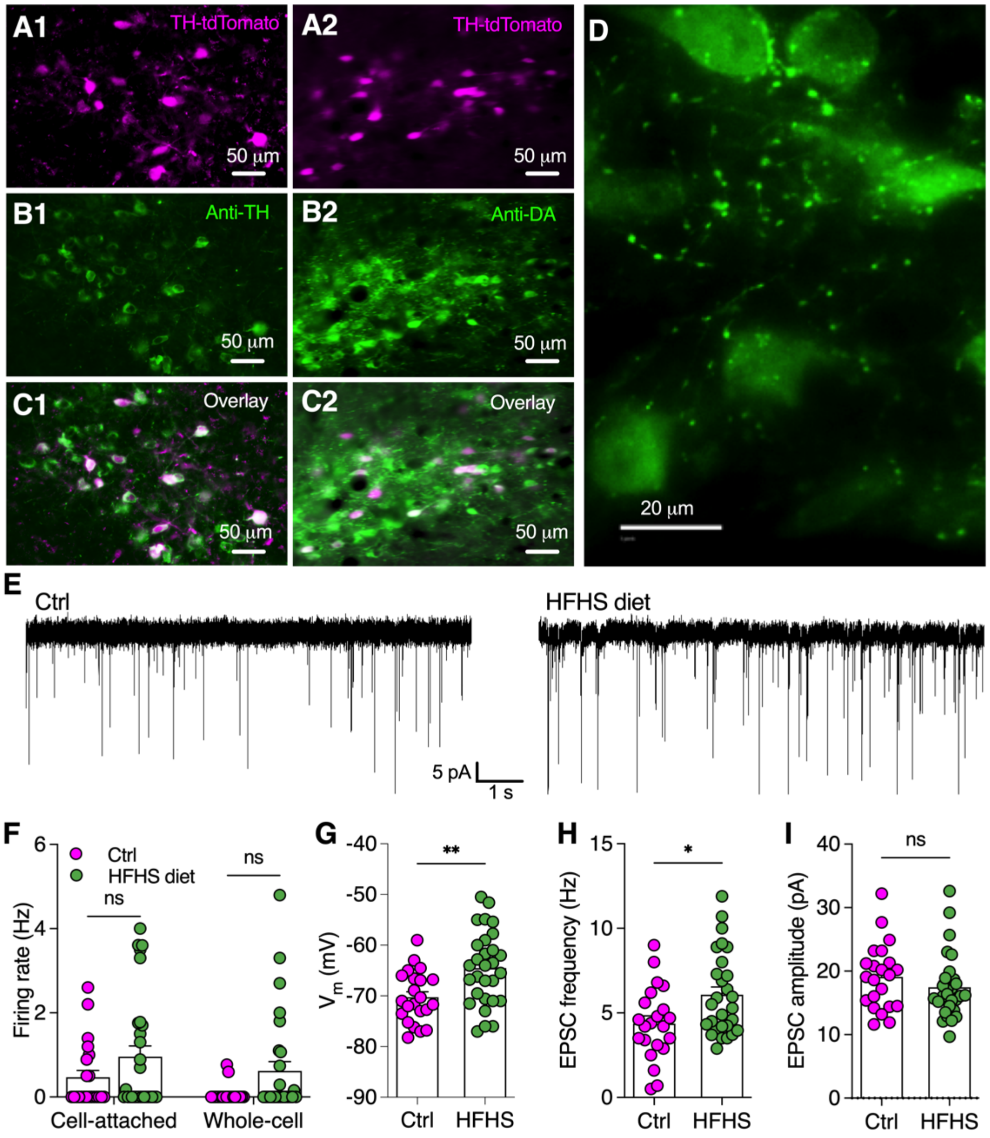

Fig. S6: HFHS diet for 2 weeks potentiated excitatory synaptic transmissions onto ZI DA for the excitability of ZI DA neurons to increase the excitability of these neurons. (A1-A3) Representative images showing TH-immunoreactive neurons in ZI of TH-tdTomato mice. (**B1-B3**) Representative images showing DA-immunoreactive neurons in ZI of TH-tdTomato mice. (**C**) A zoomed-in image shows both DA-positive soma and axons in ZI. (**D**) Representative traces showing the spontaneous EPSCs in ZI DA neurons from a control mouse and another mouse with HFHS diet. (**E**) A bar graph with scattered data plots showing firing rate of ZI DA neurons from both control and HFHS-diet mice recorded in both cell-attached and whole-cell modes. Two-way ANOVA. (**F**) Membrane potentials of ZI DA neurons from both control and HFHS-diet mice. Unpaired t test. (**G**) sEPSC frequencies of ZI DA neurons from both control and HFHS-diet mice. Unpaired t test. (**H**) sEPSC amplitudes of ZI DA neurons from both control and HFHS-diet mice. Unpaired t test.

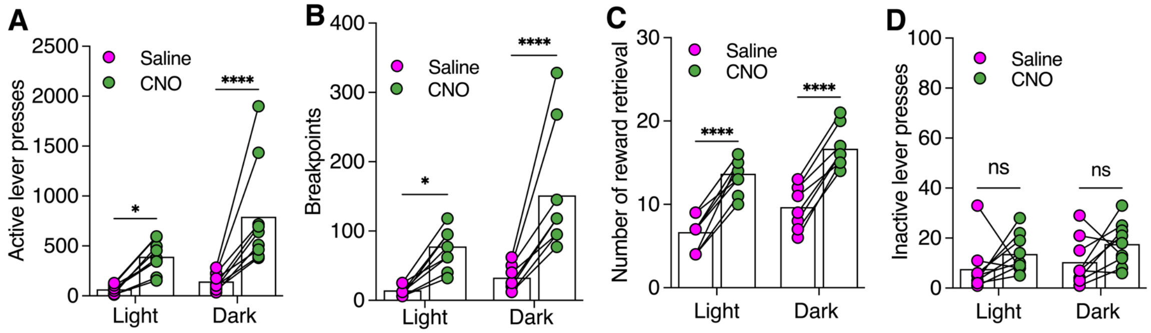

Fig. S7: Chemogenetic activation of ZI DA neurons increased active lever presses for food reward but not inactive lever presses during 45-min PR sessions with a stronger effect on tested in dark cycles. n= 9 mice. Two-way RM ANOVA with Post hoc Bonferroni test.

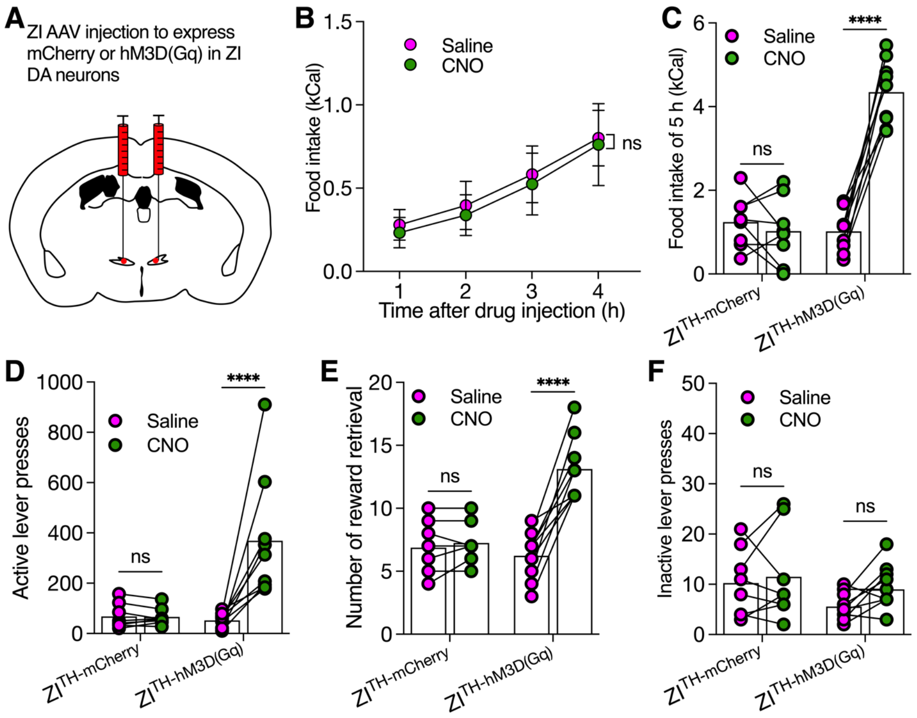

Fig. S8: Comparison of CNO effect on both food intake and food seeing in ZI$^{TH-mCherry}$ and ZI$^{TH-hM3D(Gq)}$ mice. (**A**) A diagram showing AAV5-hSyn-DIO-mCherry or AAV5-hSyn-DIO-hM3D(Gq)-mCherry was injected into bilateral ZI of TH-Cre mice. (**B**) IP injection of CNO had no effect on regular food intake over 4-h test following saline or CNO injection. Two-way RM ANOVA with Post hoc Bonferroni test. n= 8 mice. (**C**) IP injection of CNO significantly increased food intake over 5 h in ZI$^{TH-hM3D(Gq)}$ (n= 9) but not ZI$^{TH-mCherry}$ mice (n= 8). Two-way RM ANOVA with Post hoc Bonferroni test. (**D** and **E**) IP injection of CNO increased active lever presses and number of rewards earned during PR sessions of 45 min in ZI$^{TH-hM3D(Gq)}$ (n= 9) but not ZI$^{TH-mCherry}$ mice (n= 8). Two-way RM ANOVA with Post hoc Bonferroni test. (**F**) IP injection of CNO had no effect on inactive lever presses in both ZI$^{TH-hM3D(Gq)}$ (n= 9) and ZI$^{TH-mCherry}$ mice (n= 8). Two-way RM ANOVA with Post hoc Bonferroni test.

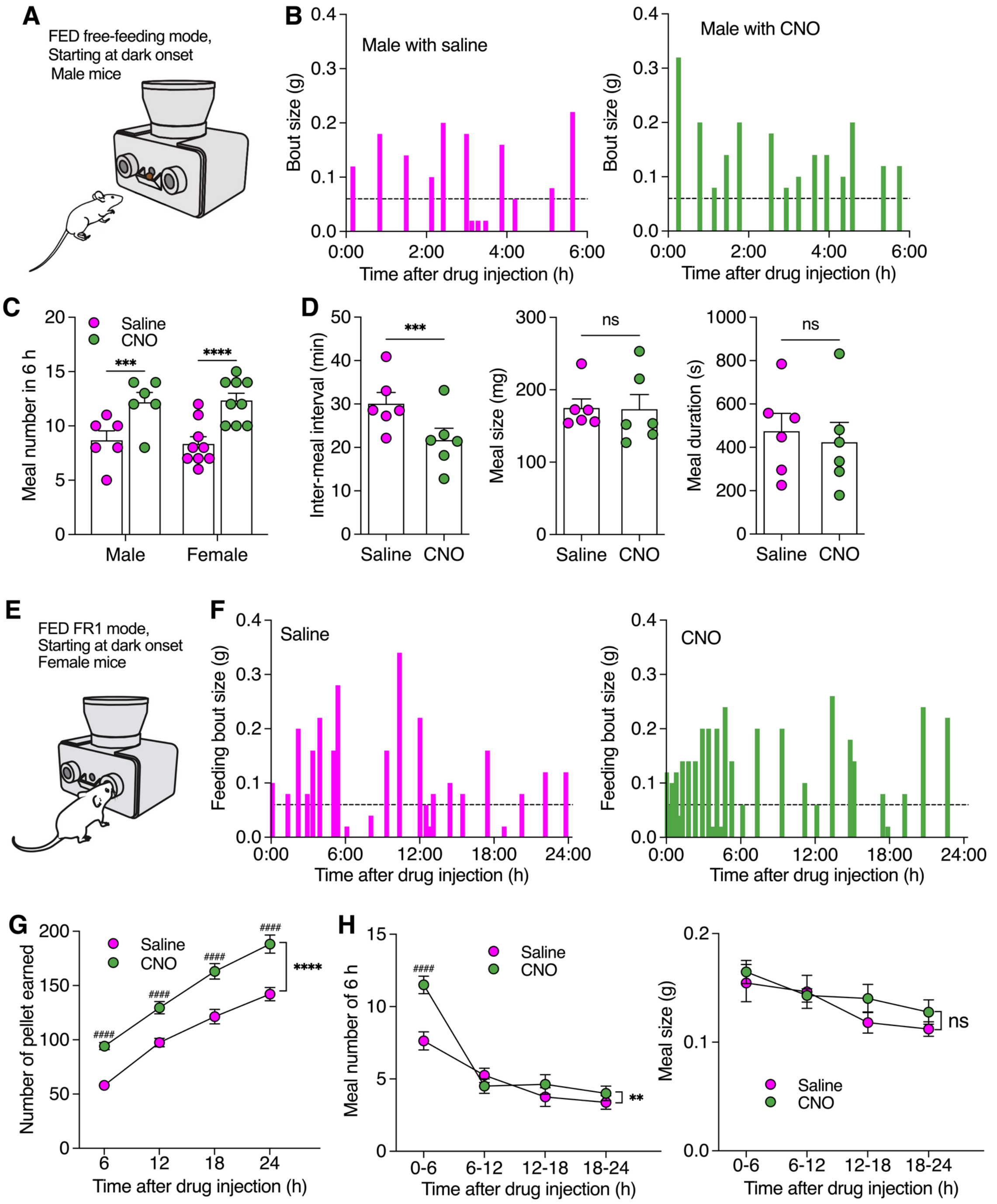

Fig. S9: Chemogenetic activation of ZI DA neurons increased meal frequency but not meal size similarly in male and female mice tested with free-feeding and FR1 operant modes using FED devices. (**A**) A diagram showing FED device for free feeding test with regular food (20 mg per pellet) starting at dark onset. (**B**) Bar graphs showing real-time feeding bout sizes following IP injection of saline and CNO (2.0 mg/kg) in a male TH-Cre mouse with hM3D(Gq)-mCherry expression in ZI DA neurons. (**C**) Meal numbers of 6 h in both male (n= 6) and female (n= 9) mice following saline or CNO injection. Two-way

RM ANOVA with Post hoc Bonferroni test. (**D**) Inter-meal intervals (left), meal size (middle), and meal duration (right) following saline or CNO injection. n = 6 male mice each group. Paired t test. (**E)** A diagram showing FED device under a FR1 mode with regular food (20 mg per pellet) starting at dark onset. One pellet was delivered immediately after each nose poke. (**F**) Bar graphs showing real-time feeding bout sizes following IP injection of saline and CNO (2.0 mg/kg) in a male TH-Cre mouse with hM3D(Gq)-mCherry expression in ZI DA neurons. (**G**) Number of pellets earned during a test duration of 24 h. n= 8 mice. Two-way RM ANOVA with Post hoc Bonferroni test. (**H**) Meal number (left) and averaged meal size (right) per 6 h following IP injection of saline or CNO. n= 8 mice each group. Two-way RM ANOVA with Post hoc Bonferroni test.

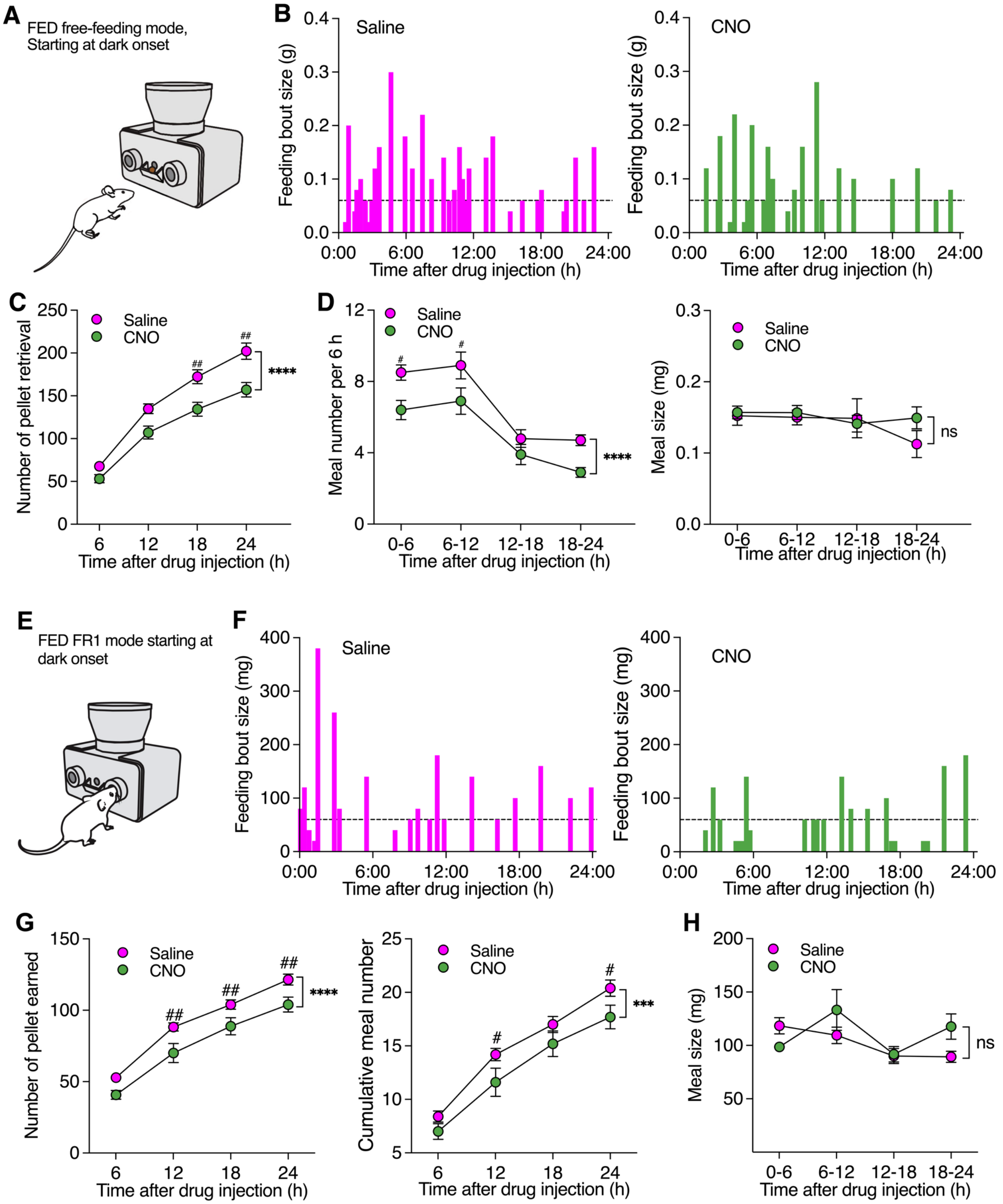

Fig. S10: Chemogenetic inhibition of ZI DA neurons decreased meal frequency but not meal size tested with free-feeding and FR1 modes using FED devices. (**A**) A diagram showing FED device under free-feeding mode with regular food (20 mg per pellet) starting at dark onset. (**B**) Bar graphs showing real-time feeding bout sizes following IP injection of saline and CNO (2.0 mg/kg) in a male TH-Cre mouse with hM4D(Gi)-mCherry expression in ZI DA neurons. (**C**) Number of pellet retrieval during a test duration of 24 h.

n= 10 mice. Two-way RM ANOVA with Post hoc Bonferroni test. (**D**) Meal number (left) and averaged meal size (right) per 6 h following IP injection of saline or CNO. n= 10 mice each group. Two-way RM ANOVA with Post hoc Bonferroni test. (**E**) A diagram showing FED device under a FR1 mode with regular food (20 mg per pellet) starting at dark onset. (**F**) Bar graphs showing real-time feeding bout sizes following IP injection of saline and CNO (2.0 mg/kg) in a male TH-Cre mouse with hM4D(Gi)-mCherry expression in ZI DA neurons. (**G**) Cumulative number of pellets earned (left) and meal number (right) over 24 h following IP injection of saline or CNO. n= 10 mice. Two-way RM ANOVA with Post hoc Bonferroni test. (**H**) Averaged meal size (right) per 6 h following IP injection of saline or CNO. n= 10 mice each group. Two-way RM ANOVA with Post hoc Bonferroni test.

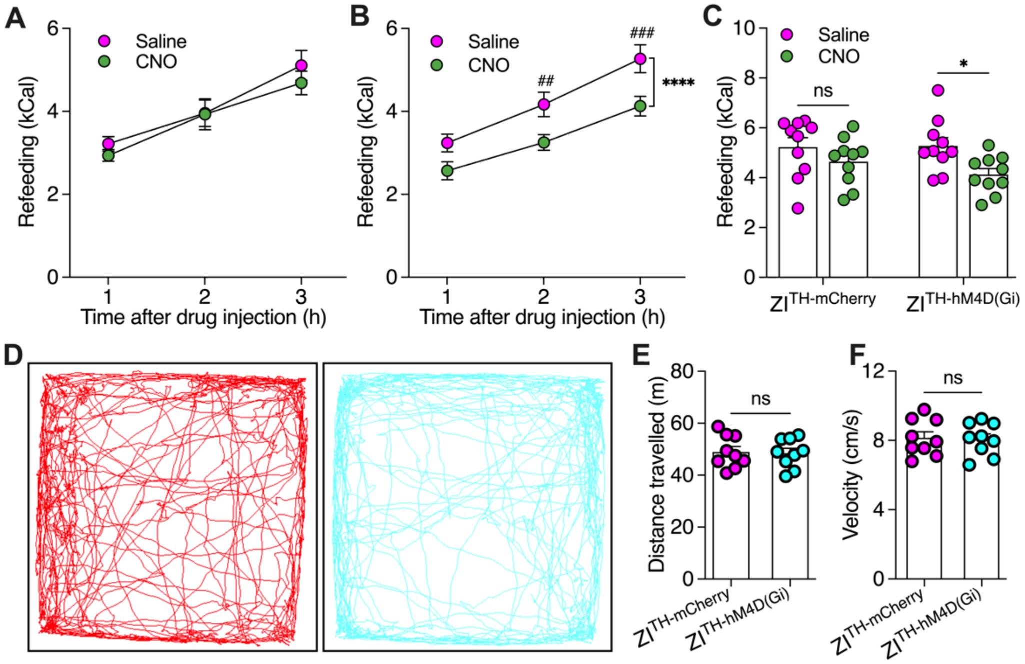

Fig. S11 Comparison of CNO effects on both $ZI^{TH-mCherry}$ and $ZI^{TH-hM4D(Gi)}$ mice. (**A**) CNO IP injection had no effect on refeeding over 3 h after 24 h fasting in control $ZI^{TH-mCherry}$ mice (n= 10). (**B**) CNO IP injection significantly reduced refeeding over 3 h after 24 h fasting in $ZI^{TH-hM4D(Gi)}$ mice (n= 10). (**C**) A bar graph with data plots showing CNO effects on refeeding over 3 h after fasting in both $ZI^{TH-mCherry}$ and $ZI^{TH-hM4D(Gi)}$ mice (n= 10 each group). (**D**) Real-time activity of one well-fed $ZI^{TH-mCherry}$ (left) and another $ZI^{TH-hM4D(Gi)}$ (right) mouse in open-field chambers after CNO IP injection. (**E**) Total of distance travelled in open-field chambers. n= 9 mice each group. Unpaired t test. (**F**) Velocity of mice travelled in open-field chambers. n= 9 mice each group. Unpaired t test.

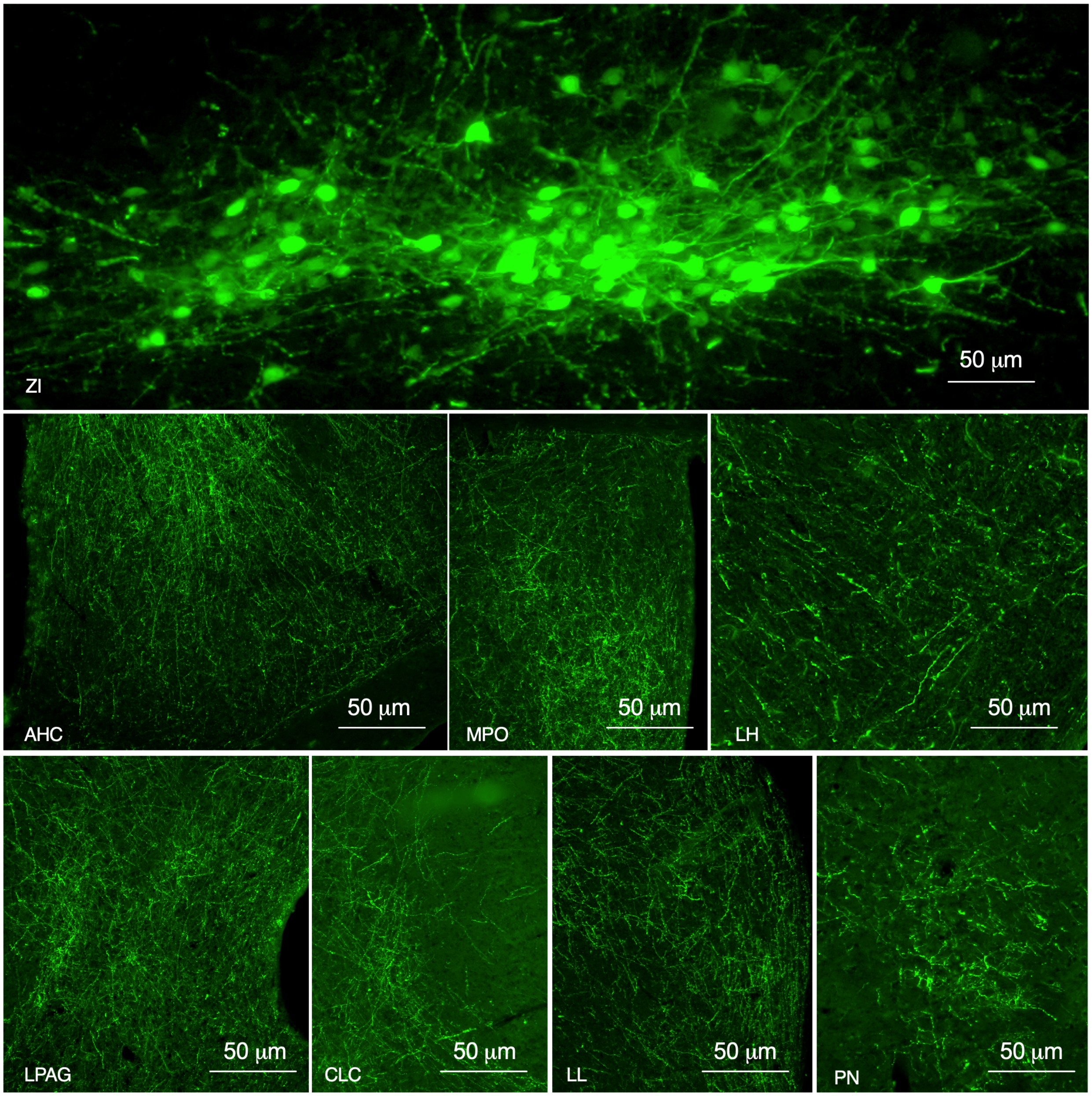

Fig. S12: Other major brain regions targeted by ZI DA neurons. AHC: anterior hypothalamus, central part**;** CLC: central nucleus of inferior colliculus; LH: lateral hypothalamus; LL: lateral lemniscus; LPAG: lateral periaqueductal gray; MPO: medial preoptic nucleus; PN: pontine nucleus; ZI: zona incerta.

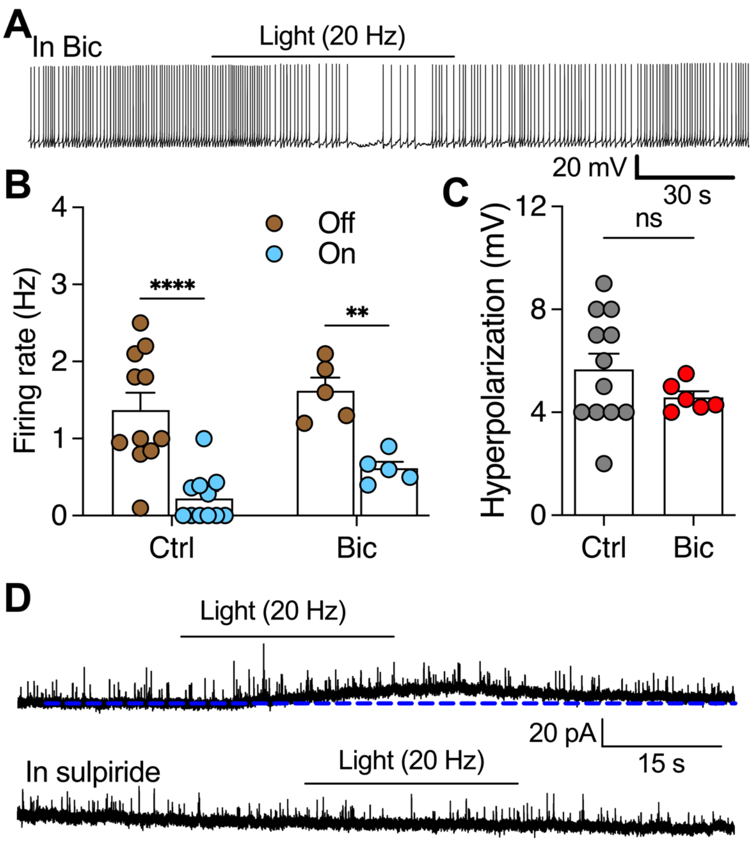

Fig. S13: Photostimulation of ZI DA terminals in the PVT inhibited PVT neurons mainly by D2 receptor and partially by $GABA_A$ receptor activation. (**A**) A representative trace showing light-induced inhibition of PVT neuron innervated by ChR2-positive ZI DA terminals in the presence of Bic (30 μM). (**B**) The firing rates of PVT neurons before and during photostimulation in the control condition and in the presence of Bic. (**C**) The membrane hyperpolarization of PVT neurons in the control condition and in the presence of Bic. (**D**) Representative traces showing a selective D2 receptor antagonist sulpiride (10 μM) abolished photostimulation-evoked tonic outward currents in PVT neurons.

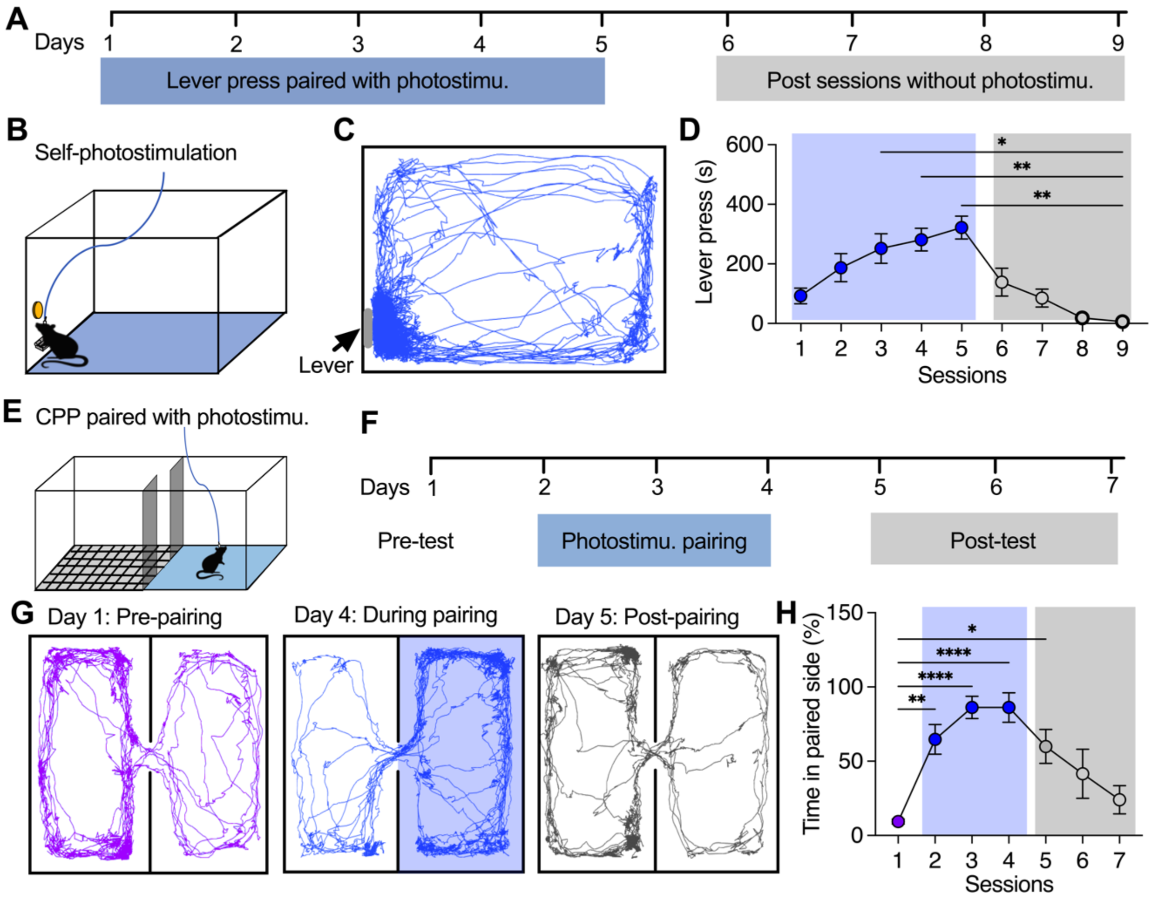

Fig. S14: Activation of ZI-PVT DA projections evoked positive valence and promoted reward memory. (**A**) A schematic diagram showing the experimental process for self-photostimulation of ZI-PVT DA terminals. (**B**) An operant chamber was used for self-photostimulation training and test. Mice with ChIEF expression in ZI DA neurons and fiber optics targeting PVT were used for the experiments. Photostimulation (20 Hz) was activated when lever was pressed down. (**C**) A representative real-time motion track illustrates a mouse spent most of the time near the lever for evoking photostimulation. (**D**) The cumulative lever-press time during a 30-min daily test session were recorded for 9 days. The first 5 sessions were paired with photostimulation of 20 Hz when the lever was pressed down, while the last 4 test sessions were paired with a sham stimulation. One-way RM ANOVA with Post hoc Bonferroni test. (**E**) A diagram showing mice were placed in a two-compartment chamber for conditioned place preference test. One compartment was grounded with mess grids and another one remained smooth. The smooth compartmental side was paired with photostimulation (20 Hz). (**F**) A schematic diagram showing the experimental process for pre-test without photostimulation, photostimulation-paired trainings, and post-test without photostimulation. (**G**) Representative tracks show the real-time motion of a $ZI^{TH\text{-}ChIEF\text{-}tdTomato}$ mouse in a two-compartment chamber before (day 1), during (day 4 of pairing), and post-paring test (day 5). (**H**) Percentages of time that mice spent in compartmental side designed with photostimulation before, during and post photostimulation paring. n= 5 mice each group. One-way RM ANOVA with Post hoc Bonferroni test.

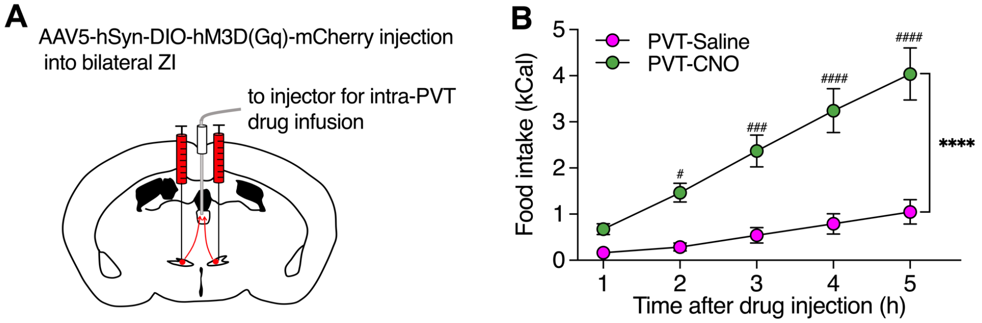

Fig. S15: Chemogenetic activation of ZI DA axonal terminals in PVT increased food intake. (**A**) A diagram showing AAV5-hSyn-DIO-hM3D(Gq)-mCherry was injected into bilateral ZI of TH-Cre mice and a canular was implanted to target PVT. (**B**) Food intake over 5 h after infusion of saline or CNO (0.5 mM, 0.5 μL) into PVT.

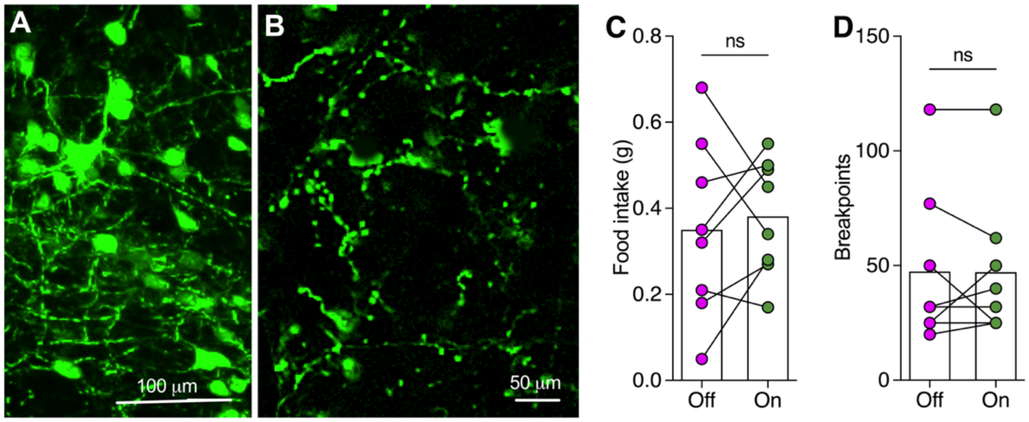

Fig. S16: Optogenetic activation of ZI-PAG DA projections has no effect on food intake. (**A**) EYFP-positive DA neurons were found in ZI when AAV1-EF1a-DIO-ChR2(H134R)-EYFP-WPRE-HGHpA was injected into ZI of TH-Cre mice. (**B**) EYFP-positive DA terminals were detected in PAG. (**C**) Photostimulation (20 Hz) of ZI-PAG DA projections had no effect on HFHS intake. (**D**) Photostimulation of ZI-PAG DA projections had no effect on breakpoints during PR sessions of 45 min.

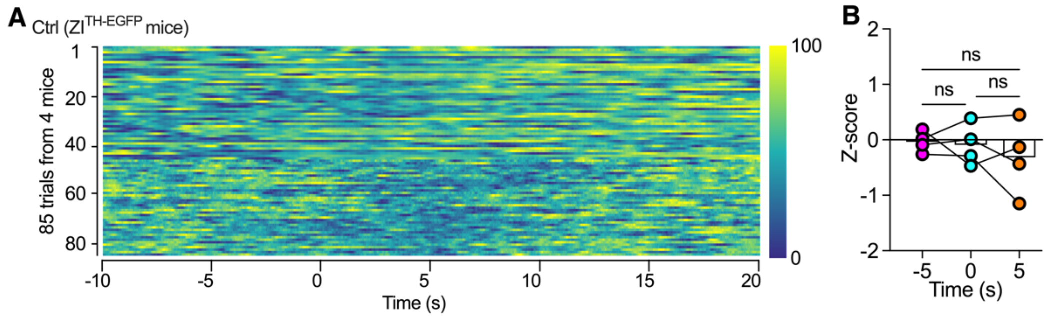

Fig. S17 Fiber-photometry imaging in control ZI$^{TH\text{-}EGFP}$ mice during food approach and consumption. (**A**) Heatmap of normalized Z-scores of ZI DA neuronal imaging from 85 feeding trials of ZI$^{TH\text{-}EGFP}$ mice (n= 4). (**B**) A bar graph with data plots showing no difference of z-score at 5 s before food approach, during approach, and during food consumption in 5 s following food approach.

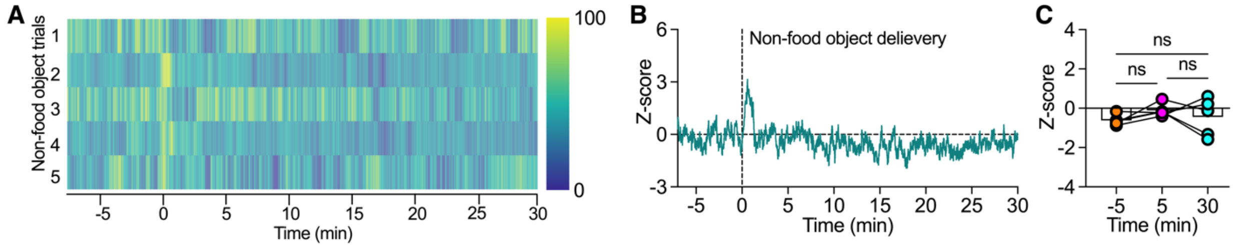

Fig. S18 Experiments with non-food object for refeeding control. (**A**) Heatmap of normalized z-scores of ZI DA neuron activity from 5 refeeding control trials with non-food object in 5 fasted ZI$^{TH\text{-}GCaMP}$ mice. (**B**) Z-scores of ZI DA neurons aligned to the time of non-food object delivery from 5 fasted ZI$^{TH\text{-}GCaMP}$ mice. (**C**) Bar graph with data plots showing the averages of z-score at the time of 5 min before refeeding, 5 and 30 min after non-food object delivery (n= 5 mice).
